# Sampling Plant Diversity and Rarity at Landscape Scales: Importance of Sampling Time in Species Detectability

**DOI:** 10.1371/journal.pone.0095334

**Published:** 2014-04-16

**Authors:** Jian Zhang, Scott E. Nielsen, Tess N. Grainger, Monica Kohler, Tim Chipchar, Daniel R. Farr

**Affiliations:** 1 Department of Renewable Resources, University of Alberta, Edmonton, Alberta, Canada; 2 Department of Ecology and Evolutionary Biology, University of Toronto, Toronto, Ontario, Canada; 3 Alberta Biodiversity Monitoring Institute, Edmonton, Alberta, Canada; University of Vigo, Spain

## Abstract

Documenting and estimating species richness at regional or landscape scales has been a major emphasis for conservation efforts, as well as for the development and testing of evolutionary and ecological theory. Rarely, however, are sampling efforts assessed on how they affect detection and estimates of species richness and rarity. In this study, vascular plant richness was sampled in 356 quarter hectare time-unlimited survey plots in the boreal region of northeast Alberta. These surveys consisted of 15,856 observations of 499 vascular plant species (97 considered to be regionally rare) collected by 12 observers over a 2 year period. Average survey time for each quarter-hectare plot was 82 minutes, ranging from 20 to 194 minutes, with a positive relationship between total survey time and total plant richness. When survey time was limited to a 20-minute search, as in other Alberta biodiversity methods, 61 species were missed. Extending the survey time to 60 minutes, reduced the number of missed species to 20, while a 90-minute cut-off time resulted in the loss of 8 species. When surveys were separated by habitat type, 60 minutes of search effort sampled nearly 90% of total observed richness for all habitats. Relative to rare species, time-unlimited surveys had ∼65% higher rare plant detections post-20 minutes than during the first 20 minutes of the survey. Although exhaustive sampling was attempted, observer bias was noted among observers when a subsample of plots was re-surveyed by different observers. Our findings suggest that sampling time, combined with sample size and observer effects, should be considered in landscape-scale plant biodiversity surveys.

## Introduction

Species richness, defined as the total number of species present in a given area, is the single most widely used measure of diversity in a biological community. Documenting and understanding patterns of species richness of different taxa at local, regional and global scales remains a major challenge within the fields of conservation biology, biogeography and macroecology, despite the large number of existing studies (e.g., [1]–[3]). This is in part because of the challenges involved in estimating species diversity at larger scales [4] or even at a regional scale [5]–[7]. Given rapidly changing climates and land use patterns, sampling and knowledge of species patterns has become increasingly important [8]. Estimating species richness is ultimately a sampling problem [9]. In practice, incomplete sampling and sampling bias are the norm [10]–[13], because not all species present may be detected. Failing to account for incomplete detection could result in false absences, leading to biases in estimates of biodiversity, species distribution, population size, survival and recruitment rates, and management decisions for rare or threatened species [14], .

Probability of species detection can be increased by increasing sampling effort, such as sample size, plot size (the area of each sample), sampling time, and the number and ability of observers [16]–[18]. This increases, however, the cost of field surveys and data collection [19]. For regional or landscape-scale diversity sampling, a balance between sampling effort and the cost in time and resources is needed. For plants, a number of studies have tested possible effects of different sampling efforts on the effectiveness of field surveys. For example, Chen *et al.* [20] examined how different factors, including sample size, plot size, observers and plant morphology, affected species detection of six woody plant species in a subtropical forest, and found that detection probability was strongly related with sample size and plot size. Investigations into the effect of observer bias on plant species detection, Nilsson & Nilsson [16], Scott & Hallam [21] and Archaux *et al.* [17] found that a single observer could miss on average 10% to 30% of the species. Alexander *et al.* [15] found that single or pairs of observers had high error rates in detecting the patches of one rare plant species, *Asclepias meadii*, at two prairie sites, while 3–4 observers generally found 90–99% of all the patches.

Sampling time, the time spent in each sample site or the time spent to detect one “new” species, is sometimes limited for regional or landscape studies or monitoring programs (e.g., [22]). However, the role of sampling time on detecting species richness and rarity is seldom considered or studied. A log-linear relationship between total sampling time and observed species richness had been previously reported (e.g., [16], [23]), but few studies on plant richness reported detailed analyses on the effects of sampling time. Archaux *et al.* [17] carried out one-hour censuses for twenty-four 400 m^2^ forest quadrats to analyze how sampling time affected observed and estimated plant richness in French lowland forests, and found that the level of exhaustiveness of plant censuses increased curvilinearly with sampling time. They also expected that many rare or endangered species that have low cover might be missed if the sampling time was limited, but didn't quantitatively assess the influences of sampling time on rare species detection. Garrard *et al.* [24], [25] proposed a novel method for estimating average time necessary to detect one or multiple plant species by considering plant traits (flower color, flowering period, and species distinctiveness) and observer experience, and applied their method to 78 plant species in 14 one-hectare grassland sites. They found that, population size, observer experience and flower color had substantial influences on average detection time [25]. Clearly, more work is needed to explore how detection of common and rare species changes as a function of sample time (effort).

In addition to recent progress in assessing the effects of different sampling efforts, the literature has focused on finding a reliable method for accurately estimating species richness (e.g., [6], [12], [26], [27]). Both the parametric and non- parametric methods for estimating species richness have been developed and compared [7], [28], and no single estimator has been shown to be superior in all situations [6]. For example, Xu *et al.* [7] used the sampling data from 164 25×25 m^2^ quadrants in a tropical forest to compare six non-parametric estimators and six parametric estimators, and found that non-parameters estimators always underestimated species richness, while parameter based estimators always overestimated species richness. Thus, more work is needed to find accurate estimators of true species richness [29]. Reliable estimation can only be obtained with both relatively complete and non-biased samples of diversity and robust estimation methods [29].

In this study, we took advantage of a large set of sample data with almost exhaustive sampling efforts and recently well-developed species richness estimators. Based 356 time-unlimited vascular plant surveys in quarter-hectare plots in the boreal forest region of northeast Alberta, Canada, we evaluated the effects of sampling time, sample size, and observer bias on measures and estimates of vascular plant species richness and rarity. In contrast to most previous studies, we recorded sampling time for each individual species' detection at each time-unlimited survey plot. Our objective was to compare and test how time-limited and time-unlimited surveys affected vascular plant species richness estimates and detection rates of rare plants. We also assessed how sample size and observer bias affect the estimates of species richness and rarity, and how the effects of sampling time and sample size vary among different vegetation types.

## Methods

### Study Area

**Figure 1:**
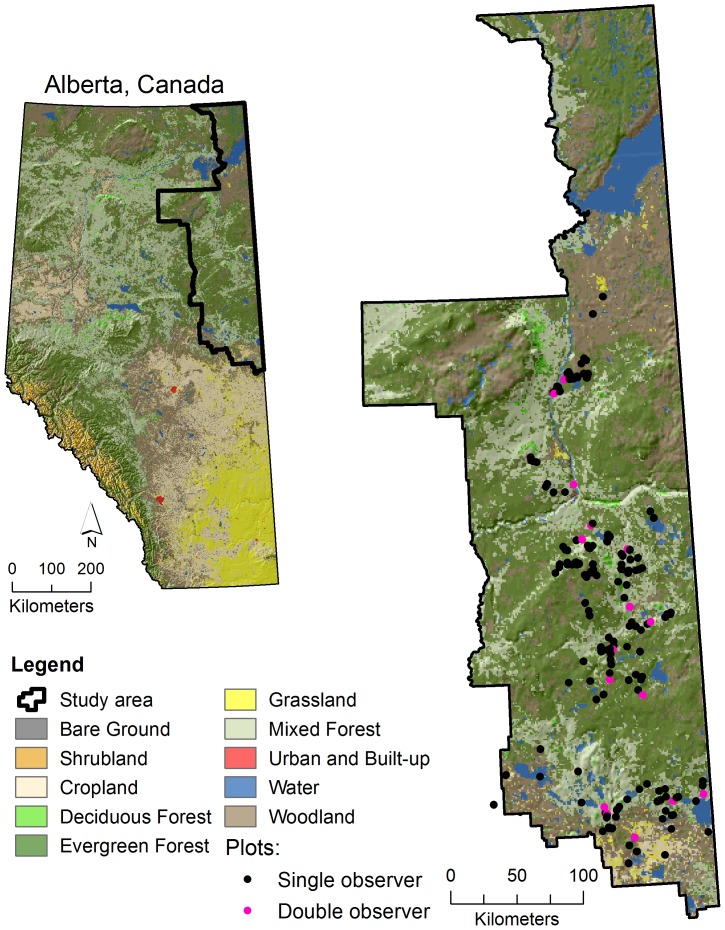
Locations of 356 sampling sites in the Lower Athabasca region of northeast Alberta. Black locations (dots) are EMCLA plots with a single survey (observer), while pink locations (dots) are EMCLA plots with repeated surveys by two field observers.

The study was conducted in the Lower Athabasca Regional Planning (LARP) area of north-eastern Alberta at 54° to 60° N latitude, 110° to 114° W longitude or approximately 93,212 km^2^ (Figure 1). Comparatively, this region is about the same size as the country of Portugal or the State of Maine. This area contains 14% of Alberta's land area, 25% of Alberta's Boreal ecosystem, and 13% of Canada's Boreal Plains ecosystem. Elevation ranges from 180 m to 820 m a.s.l. Climate is continental with mean annual temperature of −0.9°C and mean monthly temperature ranging from −26.9°C (coldest month) to 22.2 °C (warmest month). Mean annual precipitation is about 438 mm and varies across the region from 347 mm to 493 mm.

A diversity of landforms, mostly derived from the most recent glacial period, occur throughout the area affecting patterns of vegetation and species distributions. The region is characterized as being within the Boreal forest, which is dominated by deciduous, mixed wood and coniferous forests interspersed with extensive wetlands, lakes and streams, as well as unique landforms such as eolian dunes. Dominant tree species includes aspen (*Populus tremuloides*), white spruce (*Picea glauca*), black spruce (*Picea mariana*), and jack pine (*Pinus banksiana*). A wide range of plant species, wildlife and fish exist in the region, including over 500 vascular plant species and threatened woodland caribou (*Rangifer tarandus caribou*).

Much of the region consists of undeveloped forests, although substantial economic development occurs throughout the area including oil sands development, forestry and in the south small areas of agriculture. This region includes most of Alberta's bitumen deposits, which accounts for ∼10% of the world's proven oil reserves (third largest petroleum reserve in the world) and currently produces ∼1% of the global oil supply. It is expected to produce 3% of global supply by 2020 and the extraction of this resource therefore poses future threats to biodiversity. Rapid expansion of these oil sands has raised environmental concerns about managing cumulative effects on biodiversity conservation, air and water quality, and other related environmental and social issues [30].

### Time-unlimited Vascular Plant Survey

To improve the quality and consistency of wildlife and biodiversity monitoring in this region, the Ecological Monitoring Committee for the Lower Athabasca (EMCLA) was established in 2010. The EMCLA rare plants project was designed to fill existing gaps in knowledge of vascular plant distributions in the region by providing a coordinated effort of monitoring of plants to enhance detection of rare species. The project uses a stratified (habitats) and model-based (targeted) system for selecting sites for sampling. The advantage of the model-based system is that it allows for increased sampling effort in rare habitat types, while reducing effort spent sampling common habitats [31]. Whereas previous time-limited plant surveys in the region, such as the ABMI (Alberta Biodiversity Monitoring Institute), restricted the amount of time surveying plants to 20 minutes per quarter hectare plot [22], [32], EMCLA protocols provided technicians with unlimited time within quarter hectare plots to presumably increase detection rates of rare species on a per unit area basis.

Surveys were conducted in July and August of 2012 and 2013. Plot size was 50×50 m (0.25 ha). Plots were positioned to avoid roads, have less than 25% of their area affected by current or past human disturbance, and have maximum potential for finding rare plants (e.g. on open sand, rock faces, ephemeral habitats, or in transition zones between habitats) [33]. Each plot was surveyed for vascular plants by a technician capable of identifying more than 80% of species encountered with unknown plants collected for future identification. Starting in the northwest corner of the plot, the technician searched for plants while walking in a pattern that mimics a series of 50 m parallel belt transects where technicians scan a 2 to 4 m wide (1–2 m per side) strip. Each new species observed was recorded, along with the time of discovery. Unknown species were collected for later identification. Searches had no time limit and were terminated when the technician had surveyed thoroughly the entire area. Species still unidentified after the use of keys and taxonomic guides following plot completion were pressed, labeled with a unique identifier and sent to a specialist at the Royal Alberta Museum for identification. For all locations used in this study, no specific permission was required, and no specific locations of species distribution were mentioned. All data will be available to the public at the EMCLA website (http://www.emcla.ca).

To assess species detectability and observer bias between field technicians, 36 of the 356 plots (∼10%) were randomly selected to be resurveyed by a second technician on the same day and without the presence of the first observer (Figure 1).

### A Direct Comparison with an Existing Regional Monitoring Program

In order to assess the efficiency of time-unlimited surveys, we used the above field survey protocol to record vascular plant richness in four randomly selected ABMI one-hectare plots each with four quarter-hectare quadrats. ABMI was founded in 2003, and is the most influential biodiversity monitoring program in Alberta. ABMI is designed to monitor species diversity for a select group of taxa (mammals, birds, mites, vascular plants, lichens, and bryophytes) in 1,656 plots evenly spaced (20×20 km) across the province. For vascular plants, ABMI divides each one-hectare plot into four 50×50 m quadrats and limits surveys in each quarter hectare to a 20-minute search effort [22], [34]. For each of the four ABMI quadrats, both EMCLA and ABMI crews independently surveyed vascular plant richness using their own sampling protocols.

### Physical Characteristics and Ecosite Classification

Physical characteristics of each EMCLA plot were recorded. These include elevation, slope, aspect, and the type and percent cover of human and natural disturbance. Primary ecological site type (ecosite) for each plot was determined based on the dominant vegetation community and structural stage [35]. To classify an ecosite, moisture and nutrient categories were determined based on the understory plant community with a tree species modifier and structural stage assigned to the classification.

### Definition of Rarity

NatureServe has developed conservation status criteria similar to those of IUCN (the International Union for Conservation of Nature) for evaluating extinction risk at global, national and sub-national scales [36]. Criteria used by NatureServe for status assessments include abundance, range, threats to population and habitat and population trends (http://www.natureserve.org/explorer/ranking.htm). Subnational conservation status ranks (S-rank), which document the condition of the species or ecosystem within a particular state or province, were used for the current study. Specifically, we considered species to be rare if they were classified as S1 (critically imperiled), S2 (imperiled) or S3 (vulnerable).

### Data Analysis

#### Non-parametric estimators of species richness

For incidence-based sampling data, previous studies have shown that several non-parametric estimators (e.g., Chao's incidence-based estimator and the second-order jackknife estimator) were least biased in empirical comparisons and benchmark surveys, and had a more rigorous framework of sampling theory than parametric estimators [6], [7], [26]. Thus, we used two popular non-parametric species-richness estimators, Chao's incidence-based estimator (Chao2) [37] and second-order Jackknife estimator (Jack2) [38] to estimate the ‘true’ species richness for the whole study area and each ecosite type and characterize species richness patterns among habitats. These analyses were performed using the R package “vegan” [39].

#### Sample-based rarefaction and extrapolation

To indicate if sampling effort was sufficient to capture the species richness of the study area and ecosite types, resampling techniques were used to generate rarefaction or accumulation curves [9]. We used a sample-size-based rarefaction approach to estimate the rate of increase in species richness with increasing sample size, and then used the recently developed rarefaction-extrapolation approach to extrapolate the observed accumulation curve [12], [26]. Bootstrap methods were used to construct confidence intervals for species richness of any rarefied or extrapolated sample. All estimates were obtained by the software iNEXT (interpolation/extrapolation) [40].

#### Effects of sampling time on species richness estimation

To assess the effect of sampling time on species richness estimation, data from each plot were subsampled to simulate five possible methods that varied in the amount of time (effort) spent surveying the 0.25 ha plot. These included: (1) the first 20 minutes of survey time (only those records observed in the first 20 minutes of the survey were used) consistent with existing regional monitoring programs [32], [34]; (2) the first 40 minutes of survey time; (3) the first 60 minutes of survey time; (4) the first 90 minutes of survey time; and (5) a time unlimited survey. According to previous studies by Longino *et al.* [41] and Ellison *et al.* [42], rarefaction can be used meaningfully to compare the efficacy of different sampling methods that are used in the same area. In this study, we used rarefaction curves to compare how effective different sampling strategies were in estimating ‘true’ species richness. These analyses were performed using the software iNEXT [40]. Total and rare plant species richness for four ABMI one-hectare plots were also compared between the EMCLA time-unlimited protocols and the ABMI 20-minute survey. We also selected the first twenty minutes of data from all EMCLA surveys to mimic ABMI protocols (20 minute quarter-hectare survey) and compared these to time-unlimited surveys (EMCLA protocol) in the same plots. A paired *t* test was used to test the significance of the difference in species richness between protocols (20 minute vs. time-unlimited) with analyses performed using the R 3.0.2 software [43].

#### Observer effects on species richness estimation

We used field survey data for 36 randomly selected EMCLA plots with repeated observations to determine observer effects on measures of species richness. These plots were surveyed by two field technicians independently on the same day. In total there were 12 field technicians involved in repeated surveys. Relationships between observed richness of all plants or rare plants, and total sampling time were analyzed. We also analyzed between-observer variation in each of the 36 resurveyed plots by calculating pseudoturnover rate. The term *pseudoturnover* was introduced by Lynch & Johnson [44] to indicate sampling errors that increase apparent species turnover. Nilsson & Nilsson [16] used this term to describe false changes in species assemblages as an effect of species being missed during field surveys. If two field observers record plant species richness in one plot, observer A and B detect *S_A_* and *S_B_* species respectively, and *S_AA_* and *S_BB_* are the numbers of exclusive species for each observer, the pseudoturnover rate (*PT*) can be estimated as:



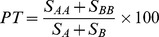
These analyses were performed using the R 3.0.2 software [43].

## Results

### Vascular Plant Species Richness

**Figure 2:**
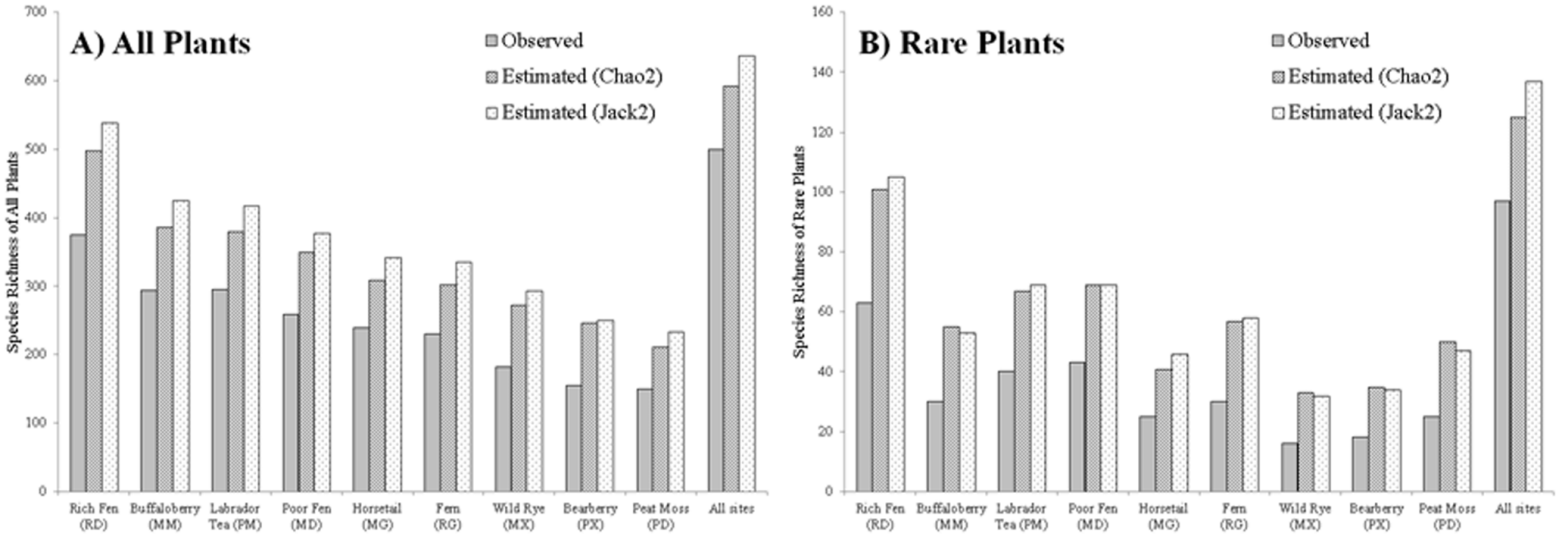
Non-parametric estimations of (A) all plant species richness and (B) rare plant species richness for the whole study area and each of 9 ecosites.

Across the 356 plots, we recorded 499 vascular plant species, 97 of which were considered regionally rare (S1, S2 or S3 status) (Figure 2, Table S1). Species belonged to 218 genera and 74 families. Species richness of vascular plants in 0.25 ha plots varied from 7 to 119, with average richness of 45 species. The most diverse ecosite was Rich Fen (RD) for which a total of 376 species were detected, followed by Labrador Tea (PM) and Buffaloberry (MM) which each had a total of 299 species.

Among 97 rare species, 8 species were S1 (critically imperiled), 7 were S2 (imperiled), and 82 were S3 (vulnerable). Rare species richness per plot ranged from 0 to 11 species. There were 35 (9.8%) plots for which no rare species were detected. Rich Fen (RD) included 64 rare species, accounting for 66% of the regional rare species richness. Poor Fen (MD) and Labrador Tea (PM) ecosites included 43 and 40 rare species, respectively.

### Non-parametric Estimation of Species Richness

Estimated species richness was 591 for the Chao2 estimator and 635 for the Jack2 estimator (Figure 2). Estimated rare species richness was 125 for the Chao2 estimator and 137 for the Jack2 estimator. Differences between observed and estimated richness varied among ecosites (Figure 2). Percent of observed total plant richness relative to Chao2 estimators ranged from 62% to 76% among ecosites. For rare plant richness, the percentages ranged from 48% to 62%.

### Comparing Time-limited and Time-unlimited Methods

**Figure 3:**
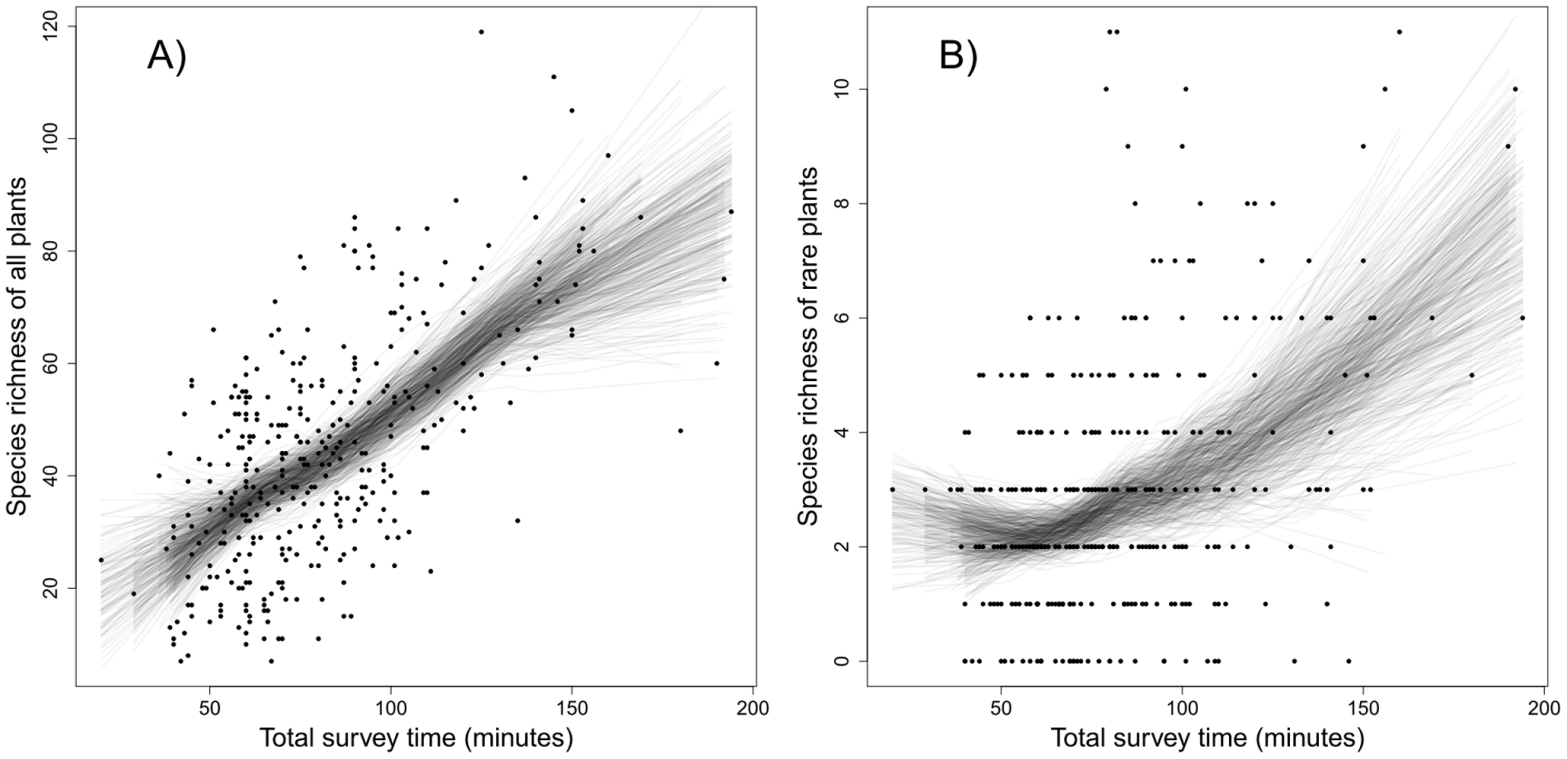
Effect of total survey time on (A) all plant richness and (B) rare plant richness using LOWESS (locally weighted scatter plot smoothing).

Across the 356 plots, average survey time per plot was 82 minutes, ranging from 20 to 194 minutes. A positive relationship was observed between total survey time of each plot and total plant richness (Figure 3A; Pearson's correlation coefficient: 0.62, p-value<0.0001).

**Figure 4:**
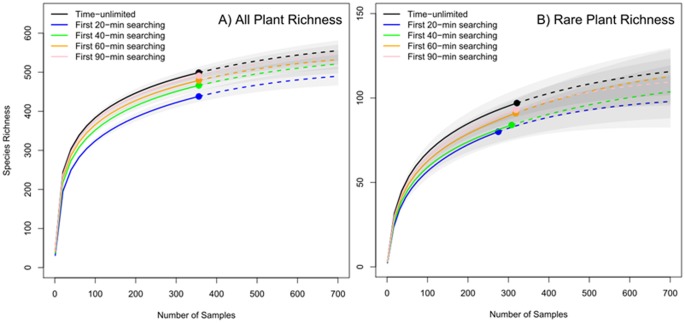
Rarefaction-based species accumulation curves for (A) all species and (B) rare species for the whole study region. Solid curves were the interpolated rarefaction curve from the reference sample. Dashed curves were the extrapolation. Each dot stands for the observed sample number.

Species incidence-based rarefaction curves present the accumulation of new species recorded in surveys over a set number of sites for five-levels of sampling effort based on sampling time (Figures 4, S1 & S2). Compared with time-unlimited surveys, ending searches at 20-min resulted in a total of 61 missed species across all surveys, while 40-min searches had a total of 33 missed species, 60-min searches had a total of 20 missed species, and 90-min searches had a total of 8 missed species (Figure 4A). A similar trend occurred for the extrapolated diversity estimates.

**Figure 5:**
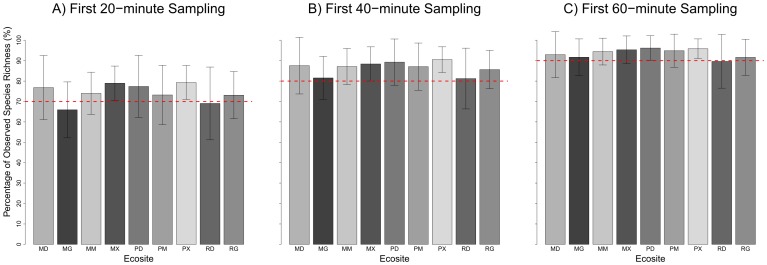
Percent of sampled species richness along different sampling time limitations: (A) first 20-minute sampling, (B) first 40-minute sampling, and (C) first 60-minute sampling.

When the EMCLA plots were separated by ecosites (Figure 5, Figure S1), ending surveys at twenty minutes also resulted in missed species. Sixty minutes of search effort sampled ∼90% of total observed richness in all ecosites (Figure 5) and gave better estimates in most ecosites (Figure S1). Larger sample sizes were needed to get better species richness estimates for several ecosites, including Horsetail (MG), Wild Rye (MX), Fern (RG), Bearberry (PX) and Peat Moss (PD) (Figure S1).

### Impact of Sampling Time on Detections of Rare Species

A significant positive relationship between total survey time and rare plant richness was detected (Figure 3B; Pearson's correlation coefficient: 0.40, p-value<0.0001).

Rare species detection rates for EMCLA plots were calculated using two time intervals: species recorded in the first 20 minutes and species recorded after the initial 20 minutes of survey (Table 1). Rare species detected after 20 minutes (9.0% of plant observations) was ∼65% higher than that of the first 20 minutes of the survey (5.8% of plant observations). Probability of detecting rare species after 20 minutes was always higher than the first 20 minutes of the survey, except for the driest ecosite (Bearberry, PX) (Table 1).

**Table 1:** Total species and number of rare species surveyed in EMCLA plots using two time intervals (20 minute cut-off and post-20 minutes).

Ecosite	# Observations≤20 minutes			# Observations>20 minutes		
	All species	Rare species	Rate of rarity (%)	All species	Rare species	Rate of rarity (%)
**Rich Fen (RD)**	2226	184	8.27	1238	129	10.42
**Buffaloberry (MM)**	2367	58	2.45	867	61	7.04
**Labrador Tea (PM)**	1933	116	6.00	840	64	7.62
**Poor Fen (MD)**	1419	121	8.53	581	74	12.74
**Horsetail (MG)**	712	20	2.81	404	25	6.19
**Fern (RG)**	646	33	5.11	258	23	8.91
**Wild Rye (MX)**	707	36	5.09	199	13	6.53
**Bearberry (PX)**	508	44	8.66	139	11	7.91
**Peat Moss (PD)**	458	27	5.90	161	24	14.91
**All sites**	11060	645	5.83	4796	432	9.01

Compared with time-unlimited surveys (Figure 4), ending searches at 20-min resulted in a total of 17 missed rare species, while 40-min searches missed 13 rare species, 60-min searches missed 6 rare species, and 90-min searches missed 4 rare species (Figure 4B). Extrapolation curves of diversity among different sampling times showed similar trends overall and among different ecosites (Figure S2).

### Observer Effects

**Figure 6:**
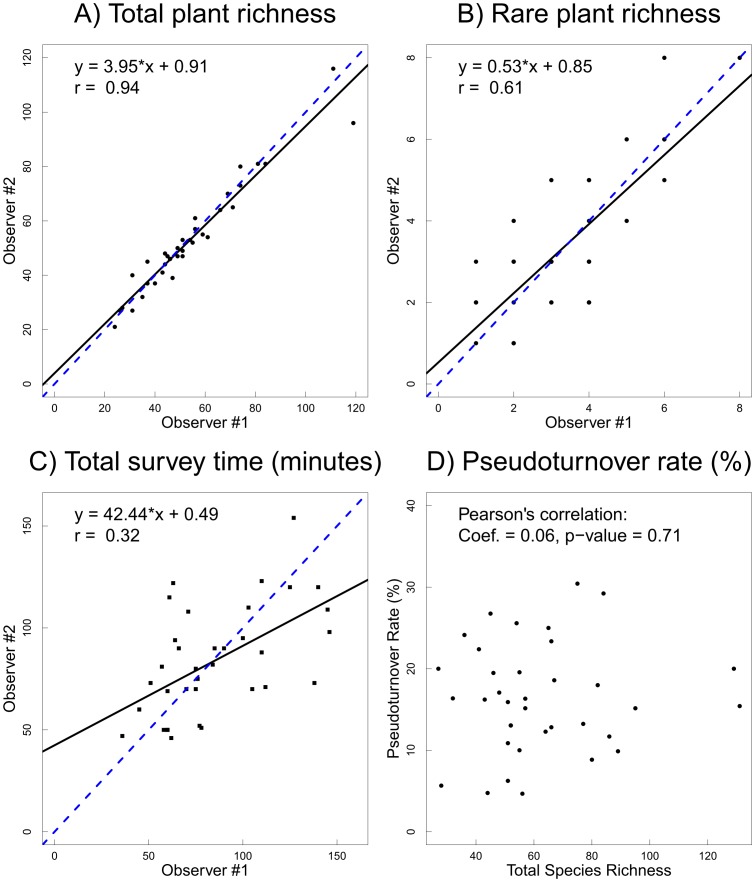
Observer effects on vascular plant richness using data from 36 EMCLA sites with repeated surveys by two observers: (A) total richness comparison, (B) rare plant richness comparison, (C) total survey time comparison, and (D) total richness *vs.* pseudoturnover rate. The solid lines were fitted by linear regression models, while the dashed lines were the 1∶1 diagonal lines that represent no bias in sampling among observers.

We compared observer bias using 36 EMCLA plots with repeated surveys (Table 2, Figure 6). Total species richness detected by two observers was very similar (Figure 6A). Differences increased in plots with higher species richness. For rare species, observer bias was larger than that of total species richness (Figure 6B). These results demonstrate that a single observer regularly missed several species, even given an unlimited amount of time in which to survey. In addition, sampling effort (time) varies substantially among observers (Figure 6C). For example at one site, one observer took one hour for the plant survey, while the other observer took nearly two hours.

**Table 2:** Summary of total vascular plant richness and rare species richness (in brackets) using the data from 36 EMCLA sites with repeated surveys by two observers.

Site ID	Total (Rare) Species Richness					Pseudoturnover Rate (%)
	Total	Observer # 1	Observer # 2	Species observed exclusively by Observer # 1	Species observed exclusively by Observer # 2	
1	54 (2)	47 (2)	39 (1)	15 (1)	7 (0)	25.58
2	46 (4)	40 (3)	37 (3)	9 (1)	6 (1)	19.48
3	32 (3)	27 (2)	28 (3)	4 (0)	5 (1)	16.36
4	43 (6)	37 (3)	37 (5)	6 (1)	6 (3)	16.22
5	55 (6)	51 (5)	49 (6)	6 (0)	4 (1)	10.00
6	57 (3)	50 (3)	49 (1)	8 (2)	7 (0)	15.15
7	51 (0)	49 (0)	47 (0)	4 (0)	2 (0)	6.25
8	51 (0)	47 (0)	45 (0)	6 (0)	4 (0)	10.87
9	28 (4)	26 (4)	27 (4)	1 (0)	2 (0)	5.66
10	64 (3)	55 (3)	59 (3)	5 (0)	9 (0)	12.28
11	82 (7)	69 (6)	70 (6)	12 (1)	13 (1)	17.99
12	86 (8)	80 (8)	74 (6)	12 (2)	6 (0)	11.69
13	27 (4)	24 (2)	21 (3)	6 (1)	3 (2)	20.00
14	48 (5)	45 (3)	37 (4)	11 (1)	3 (2)	17.07
15	80 (4)	74 (4)	73 (3)	7 (1)	6 (0)	8.84
16	89 (6)	81 (6)	81 (6)	8 (0)	8 (0)	9.88
17	66 (2)	56 (1)	61 (2)	5 (0)	10 (1)	12.82
18	84 (4)	64 (4)	66 (2)	18 (2)	20 (0)	29.23
19	65 (5)	51 (4)	53 (2)	12 (3)	14 (1)	25.00
20	66 (6)	52 (5)	55 (4)	11 (2)	14 (1)	23.36
21	36 (6)	27 (5)	31 (4)	5 (2)	9 (1)	24.14
22	41 (7)	35 (6)	32 (5)	9 (2)	6 (1)	22.39
23	51 (2)	44 (1)	44 (2)	7 (0)	7 (1)	15.91
24	52 (1)	46 (1)	46 (1)	6 (0)	6 (0)	13.04
25	55 (3)	46 (3)	46 (2)	9 (1)	9 (0)	19.57
26	129 (10)	96 (8)	119 (8)	10 (2)	33 (2)	20.00
27	67 (3)	56 (3)	57 (2)	10 (1)	11 (0)	18.58
28	57 (4)	47 (2)	51 (4)	6 (0)	10 (2)	16.33
29	77 (1)	71 (0)	65 (1)	12 (0)	6 (1)	13.24
30	131 (7)	111 (5)	116 (4)	15 (3)	20 (2)	15.42
31	56 (4)	53 (3)	54 (4)	2 (0)	3 (1)	4.67
32	95 (4)	84 (4)	81 (2)	14 (2)	11 (0)	15.15
33	45 (4)	31 (3)	40 (3)	5 (1)	14 (1)	26.76
34	75 (5)	54 (3)	61 (3)	14 (2)	21 (2)	30.43
35	44 (2)	43 (2)	41 (2)	3 (0)	1 (0)	4.76
36	52 (2)	48 (2)	44 (1)	8 (1)	4 (0)	13.04

Although total species richness varied little between two observers despite differences in amount of time sampled (Figure 6A), a number of species were detected exclusively by one observer (Table 2). For example, in site #18, the first observer detected 20 exclusive species, while the second observer detected 18 exclusive species. On average, for time-unlimited plant surveys one observer missed 8.6 species (range of 1 to 33). Overall, between-observer pseudoturnover rate averaged 16.3%, ranging from 4.7% to 30.4% (Table 2). Surprisingly, pseudoturnover rate wasn't significantly rated to total species richness (Figure 6D; Pearson's correlation coefficient: 0.06, p-value = 0.709).

Observer bias also affected rare species detections (Table 2). Among 34 plots with rare species records, 42 (61.8%) of 68 surveys had missing rare species compared with total rare species listed together by two observers.

### Comparisons with an Existing Regional Monitoring Program

A direct comparison between EMCLA and ABMI survey protocols at four one-hectare ABMI sites (16 quarter-hectare quadrats) using a 20 minute survey limit greatly underestimated total observed plant richness (Tables 3 & S2). Nearly 30–50% of plant species were not detected in the first twenty minutes of surveying like that of ABMI protocols. Significant differences in total and rare species richness were detected between ABMI time-limited surveys and EMCLA time-unlimited surveys (paired *t* tests: total richness, *t* = −8.05, *df* = 15, *P*<0.001; rare richness, *t* = −6.06, *df* = 15, *P*<0.001). Significant difference in total species richness was found between ABMI time-limited surveys and EMCLA surveys for the first twenty-minutes (paired *t* tests: *t* = −4.60, *df* = 15, *P*<0.001), but not significantly on rare species richness (paired *t* tests: *t* = −1.78, *df* = 15, *P* = 0.10).

**Table 3:** A direct comparison between EMCLA time-unlimited survey and ABMI (an existing regional biodiversity monitoring program) 20-minute survey for four one-hectare ABMI plots.

	Site ID	Total plant richness	Rare plant richness	Survey Time (hours)*^1^*
ABMI 20-minute survey	A	55	0	1.33
	B	46	2	1.33
	C	47	0	1.33
	D	52	0	1.33
EMCLA time-unlimited survey	A	107	5	6.98
	B	75	4	5.17
	C	70	2	5.88
	D	100	5	7.12
First 20-minute of EMCLA survey	A	81	1	1.33
	B	60	2	1.33
	C	59	0	1.33
	D	71	2	1.33

Note: *^1^* Total survey time for the four quadrats was used.

## Discussion

### Effects of Sampling Time on Detecting and Estimating Species Richness and Rarity

We collected 15,856 unique observations for 499 vascular plant species by 12 observers across 356 time-unlimited quarter-hectare survey plots in the boreal forests of northeast Alberta and reported the effects of sampling time, sample size and observer bias on detecting and estimating total species richness and rarity. Based on our results, total survey time spent in the field is critical for estimating species richness, especially when the focus is on species with low prevalence (e.g., rare or threaten species, or emerging invasive species), which is often the case for biodiversity conservation and monitoring efforts.

Although the importance of sampling time has been recognized by previous studies (e.g., [16], [17]), it is rarely considered in the sampling design of landscape-level biodiversity monitoring programs in both species-poor and species-rich communities. There is a tradeoff between sample size (number of sites monitored), plot size and sampling time, given budgetary and time constraints. For example, in Alberta, the ABMI (Alberta Biodiversity Monitoring Institute) is designed to monitor up 1,656 plots evenly spaced across the province to assess species diversity of select taxa. For vascular plants, the ABMI uses time-limited surveys with a 20-minute search effort for each of four quarter-hectare quadrats [22], [34]. Our analyses illustrated that a 20-minute search effort substantially underestimates species richness, particularly for rare species (Tables 1 & 3, Figure 3). We suggest that a single well-trained observer sampling a quarter-hectare site in the boreal region of northeast Alberta needs about one hour to sample 90% of total plant species (Figures 4 & 5).

The results of our non-parametric estimates of species richness indicate that time-limited survey data also underestimated species richness for the entire study region, as well as for each of the 9 ecosites sampled (Figures 4, S1 & S2). This finding strongly supports the suggestion that incomplete detection can have an important effect on documenting and estimating regional biodiversity thus potentially affecting conservation strategies and selection of priority conservation areas [13], [15]. Incomplete detection can also lead to bias in species distribution modeling [45], estimates of population size and trend [46], survival and recruitment rates [47], [48], and extinction probabilities [49]. In addition to time limitations, other factors may also cause incomplete detections, including observer experience, sample size, plant morphology, seasonal phenology, and habitat and weather conditions. Further studies are needed to integrate these factors into the sampling design of biodiversity assessments.

### Influence of Vegetation Types on Monitoring Plant Species Richness

Clearly, different vegetation types (ecosites in our study) need different survey efforts in terms of sampling time and sample size (Figures S1& S2). Previous studies have demonstrated that detection probability of species occupancy was influenced by local habitat or surrounding landscape characteristics [13], [50]. If time limits on surveys are imposed, average or minimum survey time could vary among different vegetation types and habitat categories. For measures of species diversity patterns at regional or landscape scales, pilot studies should be used to determine effort needed in each vegetation type, determine size of plots, number of samples needed, and how long the average sampling time should be if limited.

### Dealing with Observer Bias

Although the goal was to conduct exhaustive field surveys in each EMCLA plot and total species richness was similar among two observers in repeated surveys, a number of common or rare species were missed by any one single observer (Table 2, Figure 6). On average, each observer missed ∼8.6 species per plot. These results are consistent with previous studies illustrating that the observer effect is a major challenge in biodiversity surveys (e.g., [15]–[17], [23]). Previous studies have shown that average survey time is strongly influenced by observer experience [11], [25]. Although we tried to reduce this potential effect through training, survey time needed for one plant census in individual plots differed between individual observers (Figure 6C). One observer could take as much as two times longer than another to finish a plant survey for the same plot, yet resulting in similar total species richness. This finding is also supported by our comparison on plant richness detected by EMCLA and ABMI field crews. Under the same sampling time effort (20 minutes), EMCLA crews detected more species than ABMI crews in the same plot (Table 3). One main reason is that the EMCLA crew had more experience and specialized training in identifying rare plant species, while ABMI crew did not [33]. Therefore, observer bias also needs to be considered when assessing the effect of sampling time on species detection. Based on our analyses of observer bias, we believe that the ‘best’ solution to minimize errors is to do the surveys as a team [15], [23] or consider reducing plot size.

### Recommendations for Sampling Landscape-scale Plant Diversity and Rarity

Generally, plant ecologists assume that species that are present will be detected during field surveys. However, according to our results in boreal forest communities, incomplete detection of plant species richness is much more common than currently acknowledged by most plant ecologists. While the boreal region has documented lower biodiversity than temperate and tropical regions, we expect that incomplete detection of plant richness may have large effects on biodiversity surveys in species-rich communities [13], [17]. Recently, a growing body of literature has recognized this issue (e.g., [13], [20], [25], [48]). For landscape-scale sampling of plant diversity, we offer the following recommendations. First, sampling time should be an important consideration for designing biodiversity monitoring protocols with a time-unlimited survey or generous time budgets (perhaps varying by habitat). Second, observer training and working as a team may reduce observer bias. Third, pilot studies should be used to help determine optimal survey effort by considering the effects of sampling time, plot size, observer bias, plant traits, and other factors on the detection of vascular plants.

## Supporting Information

Figure S1 Rarefaction-based species accumulation curves for all vascular plant species in each of the 9 ecosites sampled.
(DOCX)

Figure S2 Rarefaction-based species accumulation curves for rare vascular plant species in each of the 9 ecosites sampled.
(DOCX)

Table S1 Vascular plant species list in 356 EMCLA plots. NatureServe subnational conservation status: S1: Critically Imperiled; S2: Imperiled; S3: Vulnerable; S4: Apparently Secure; S5: Secure; SNR: Not ranked; SNA: Not applicable; SU: currently unranked.
(DOCX)

Table S2 Comparison of observed vascular plant species richness between ABMI and EMCLA plots in each of four 0.25-hectare quadrats at four ABMI one-hectare sites.
(DOCX)

## References

[1] Brown JH (1995) Macroecology. Chicago: University of Chicago Press.

[2] Whittaker RJ (1998) Island Biogeography: Ecology, Evolution, and Conservation. Oxford: Oxford University Press.

[3] Stohlgren TJ (2007) Measuring Plant Diversity: Lessons From The Field. Oxford: Oxford University Press.

[4] Mora C, Tittensor DP, Adl S, Simpson AGB, Worm B (2011) How many species are there on Earth and in the ocean? PLoS Biol 9: e1001127 10.1371/journal.pbio.1001127 21886479 PMC3160336

[5] Hutchinson GE (1961) The paradox of the plankton. Am Nat 95: 137–145 10.1086/282171

[6] Brose U, Martinez ND, Williams RJ (2003) Estimating species richness: sensitivity to sample coverage and insensitivity to spatial patterns. Ecology 84: 2364–2377 10.1890/02-0558

[7] Xu H, Liu S, Li Y, Zang R, He F (2012) Assessing non-parametric and area-based methods for estimating regional species richness. J Veg Sci 23: 1006–1012 10.1111/j.1654-1103.2012.01423.x

[8] Thuiller W, Albert C, Araújo MB, Berry PM, Cabeza M, et al (2008) Predicting global change impacts on plant species' distributions: Future challenges. Perspect Plant Ecol Evol Syst 9: 137–152 10.1016/j.ppees.2007.09.004

[9] Gotelli N, Colwell R (2011) Estimating species richness. In: Magurran AE, McGill BJ, editors. Biological Diversity: Frontiers in Measurement and Assessment. Oxford: Oxford University Press. pp. 39–54.

[10] Beck J, Schwanghart W (2010) Comparing measures of species diversity from incomplete inventories: an update. Methods Ecol Evol 1: 38–44 10.1111/j.2041-210X.2009.00003.x

[11] Moore JL, Hauser CE, Bear JL, Williams NSG, McCarthy MA (2011) Estimating detection–effort curves for plants using search experiments. Ecol Appl 21: 601–607 10.1890/10-0590.1 21563589

[12] Colwell RK, Chao A, Gotelli NJ, Lin S-Y, Mao CX, et al (2012) Models and estimators linking individual-based and sample-based rarefaction, extrapolation and comparison of assemblages. J Plant Ecol 5: 3–21 10.1093/jpe/rtr044

[13] Chen G, Kéry M, Plattner M, Ma K, Gardner B (2013) Imperfect detection is the rule rather than the exception in plant distribution studies. J Ecol 101: 183–191 10.1111/1365-2745.12021

[14] Moore JL, Hauser CE, Bear JL, Williams NSG, McCarthy MA (2011) Estimating detection-effort curves for plants using search experiments. Ecol Appl 21: 601–607. 21563589 10.1890/10-0590.1

[15] Alexander HM, Reed AW, Kettle WD, Slade NA, Bodbyl Roels SA, et al (2012) Detection and plant monitoring programs: Lessons from an intensive survey of Asclepias meadii with five observers. PLoS One 7: e52762 10.1371/journal.pone.0052762 23285179 PMC3527611

[16] Nilsson I, Nilsson S (1985) Experimental estimates of census efficiency and pseudoturnover on islands: Error trend and between-observer variation when recording vascular plants. J Ecol 73: 65–70.

[17] Archaux F, Gosselin F, Bergès L, Chevalier R (2006) Effects of sampling time, species richness and observer on the exhaustiveness of plant censuses. J Veg Sci 17: 299–306 10.1111/j.1654-1103.2006.tb02449.x

[18] Archaux F (2009) Could we obtain better estimates of plot species richness from multiple-observer plant censuses? J Veg Sci 20: 603–611 10.1111/j.1654-1103.2009.01079.x

[19] Raven PH, Wilson EO (1992) A fifty-year plan for biodiversity surveys. Science 258: 1099–1100 10.1126/science.258.5085.1099 17789079

[20] Chen G, Kéry M, Zhang J, Ma K (2009) Factors affecting detection probability in plant distribution studies. J Ecol 97: 1383–1389 10.1111/j.1365-2745.2009.01560.x

[21] Scott WA, Hallam CJ (2003) Assessing species misidentification rates through quality assurance of vegetation monitoring. Plant Ecol 165: 101–115 10.1023/A:1021441331839

[22] Boutin S, Haughland DL, Schieck J, Herbers J, Bayne E (2009) A new approach to forest biodiversity monitoring in Canada. For Ecol Manage 258: S168–S175 10.1016/j.foreco.2009.08.024

[23] Klimeš L, Dančak M, Hájek M, Jongepierová I, Kučera T (2001) Scale-dependent biases in species counts in a grassland. J Veg Sci 12: 699–704 10.2307/3236910

[24] Garrard GE, Bekessy SA, McCARTHY MA, Wintle BA (2008) When have we looked hard enough? A novel method for setting minimum survey effort protocols for flora surveys. Austral Ecol 33: 986–998 10.1111/j.1442-9993.2008.01869.x

[25] Garrard GE, McCarthy MA, Williams NSG, Bekessy SA, Wintle BA (2013) A general model of detectability using species traits. Methods Ecol Evol 4: 45–52 10.1111/j.2041-210x.2012.00257.x

[26] Colwell RK, Coddington JA (1994) Estimating terrestrial biodiversity through extrapolation. Philos Trans R Soc B 345: 101–118 10.1098/rstb.1994.0091 7972351

[27] Chao A, Gotelli NJ, Hsieh TC, Sander EL, Ma KH, et al (2014) Rarefaction and extrapolation with Hill numbers: a framework for sampling and estimation in species diversity studies. Ecol Monogr 84: 45–67 10.1890/13-0133.1

[28] Chao A (2005) Species Richness Estimation. 2nd ed. New York: Wiley.

[29] Chiarucci A (2012) Estimating species richness: still a long way off!. J Veg Sci 23: 1003–1005 10.1111/jvs.12003

[30] Swart NC, Weaver AJ (2012) The Alberta oil sands and climate. Nat Clim Chang 2: 134–136 10.1038/nclimate1421

[31] Guisan A, Broennimann O, Engler R, Vust M, Yoccoz NG, et al (2006) Using niche-based models to improve the sampling of rare species. Conserv Biol 20: 501–511 10.1111/j.1523-1739.2006.00354.x 16903111

[32] Nielsen SE, Haughland DL, Bayne E, Schieck J (2009) Capacity of large-scale, long-term biodiversity monitoring programmes to detect trends in species prevalence. Biodivers Conserv 18: 2961–2978 10.1007/s10531-009-9619-1

[33] Nielsen SE (2013) Targeted rare plant surveys for the Lower Athabasca: A report on 2012 activities. Edmonton. Available at: http://www.emcla.ca/wp-content/uploads/2013/12/EMCLA_Rare-Plants-Report_04April2013_Revised_29Nov2013.pdf.

[34] ABMI (Alberta Biodiversity Monitoring Institute) (2010) Terrestrial field data collection protocols (10001), Version 2010-04-20. Alberta Biodiversity Monitoring Institute, Alberta, Canada. Available at: http://www.abmi.ca.

[35] Beckingham JD, Archibald JH (1996) Field Guide to Ecosites of Northern Alberta. Canadian Forest Service, Northern Forestry Centre Special Report.

[36] Faber-Langendoen D, Nichols J, Master L, Snow K, Tomaino A, et al. (2012) NatureServe Conservation Status Assessments: Methodology for Assigning Ranks. NatureServe, Arlington, VA.

[37] Chao A (1984) Nonparametric estimation of the number of classes in a population. Scand J Stat 11: 265–270.

[38] Palmer MW (1991) Estimating species richness: The second-order Jackknife reconsidered. Ecology 72: 1512–1513 10.2307/1941127

[39] Oksanen J, Blanchet FG, Kindt R, Legendre P, O'Hara RB, et al. (2013) vegan: Community Ecology Package. R Packag version 1: R package version 2.0–9. doi:10.4135/9781412971874.n145.

[40] Hsieh TC, Ma KH, Chao A (2013) iNEXT online: interpolation and extrapolation. Available at: http://chao.stat.nthu.edu.tw/blog/software-download/.

[41] Longino JT, Coddington JA, Colwell RK (2002) The ant fauna of a tropical rain forest: Estimating species richness three different ways. Ecology 83: 689–702 10.1890/0012-9658(2002)0830689:TAFOAT2.0.CO2

[42] Ellison AM, Record S, Arguello A, Gotelli NJ (2007) Rapid inventory of the ant assemblage in a temperate hardwood forest: Species composition and assessment of sampling methods. Environ Entomol 36: 766–775 10.1603/0046-225X(2007)36766:RIOTAA2.0.CO2 17716467

[43] R Core Team R (2013) R: A language and environment for statistical computing. Vienna, Austria. Available at: http://www.R-project.org.

[44] Lynch JF, Johnson NK (1974) Turnover and equilibria in insular avifaunas, with special reference to the California Channel Islands. Condor 76: 370–384 10.2307/1365812

[45] Braunisch V, Suchant R (2010) Predicting species distributions based on incomplete survey data: The trade-off between precision and scale. Ecography 33: 826–840 10.1111/j.1600-0587.2009.05891.x

[46] Alexander HM, Slade NA, Kettle WD (1997) Application of mark-recapture models to estimation of the population size of plants. Ecology 78: 1230–1237 10.1890/0012-9658(1997)0781230:AOMRMT2.0.CO2

[47] Shefferson RP, Sandercock BK, Proper J, Beissinger SR (2001) Estimating dormancy and survival of a rare herbaceous perennial using mark-recapture models. Ecology 82: 145–156 10.1890/0012-9658(2001)0820145:EDASOA2.0.CO2

[48] Kéry M, Gregg KB (2004) Demographic analysis of dormancy and survival in the terrestrial orchid Cypripedium reginae. J Ecol 92: 686–695 10.1111/j.0022-0477.2004.00885.x

[49] Kéry M (2004) Extinction rate estimates for plant populations in revisitation studies: Importance of detectability. Conserv Biol 18: 570–574 10.1111/j.1523-1739.2004.00105.x

[50] Gu W, Swihart RK (2004) Absent or undetected? Effects of non-detection of species occurrence on wildlife–habitat models. Biol Conserv 116: 195–203 10.1016/S0006-3207(03)00190-3

