# Correction: Sampling Plant Diversity and Rarity at Landscape Scales: Importance of Sampling Time in Species Detectability

**DOI:** 10.1371/journal.pone.0103920

**Published:** 2014-07-24

**Authors:** The PLOS ONE Staff

The equations in Figure 6 are incorrect. The authors have provided a corrected version here.

**Figure 6:**
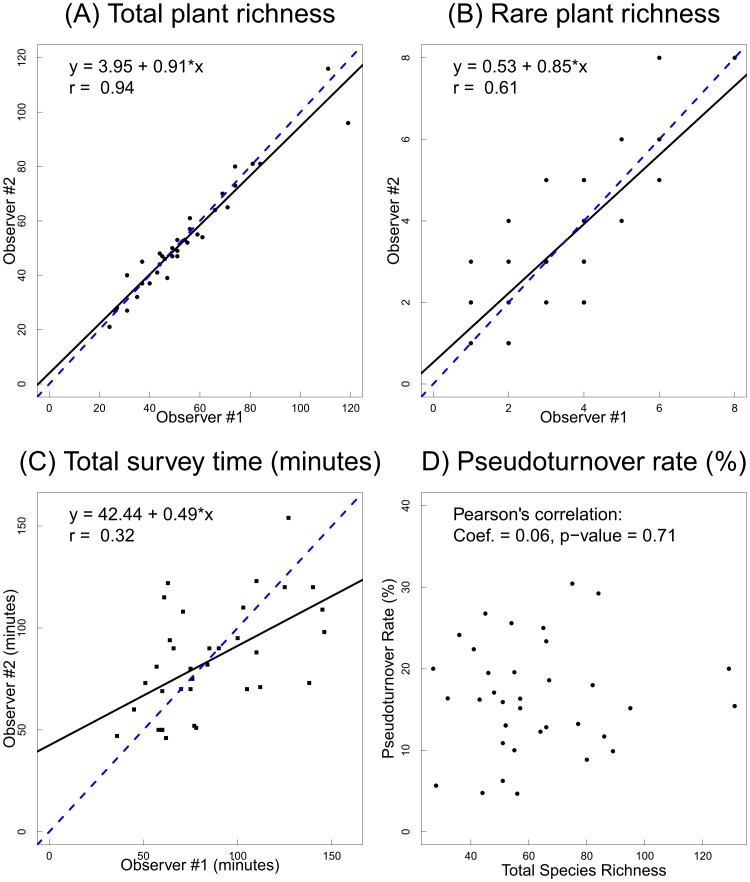
Observer effects on vascular plant richness using data from 36 EMCLA sites with repeated surveys by two observers: (A) total richness comparison, (B) rare plant richness comparison, (C) total survey time comparison, and (D) total richness *vs.* pseudoturnover rate. The solid lines were fitted by linear regression models, while the dashed lines were the 1:1 diagonal lines that represent no bias in sampling among observers.
