# Supplementary material for: Sampling Plant Diversity and Rarity at Landscape Scales: Importance of Sampling Time in Species Detectability
Source: PLoS One. 2014 Apr 16;9(4):e95334. doi: 10.1371/journal.pone.0095334 (PMC3989307; doi:10.1371/journal.pone.0095334)

## Figure S2. Rarefaction-based species accumulation curves for rare vascular plant species in each of the 9 ecosites sampled.


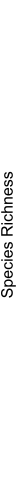

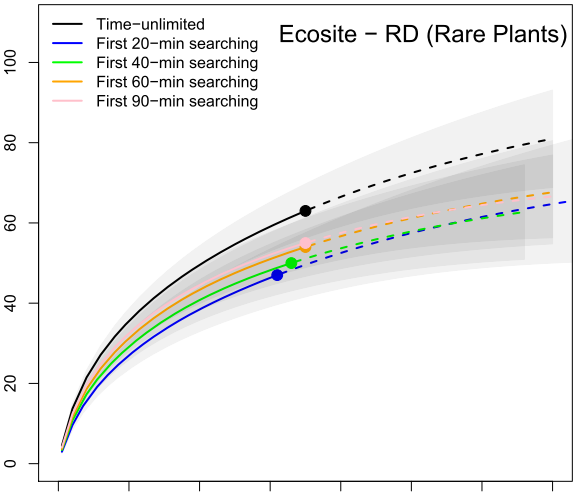

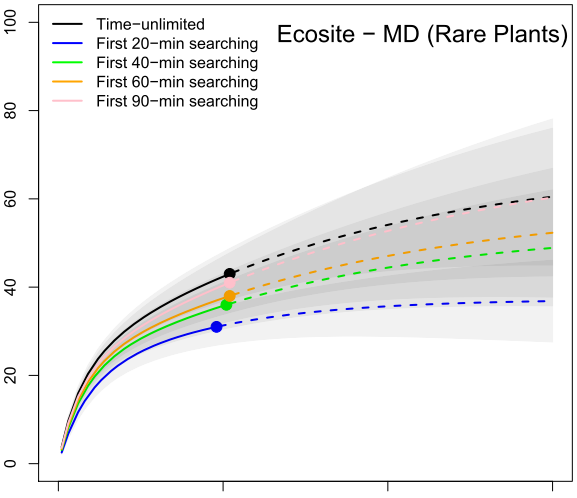

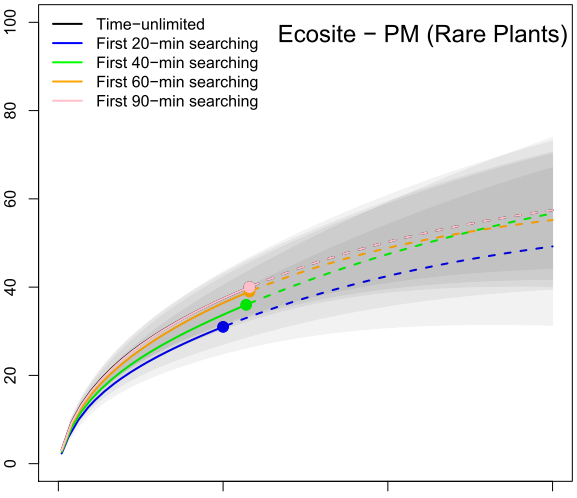

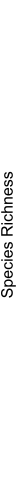

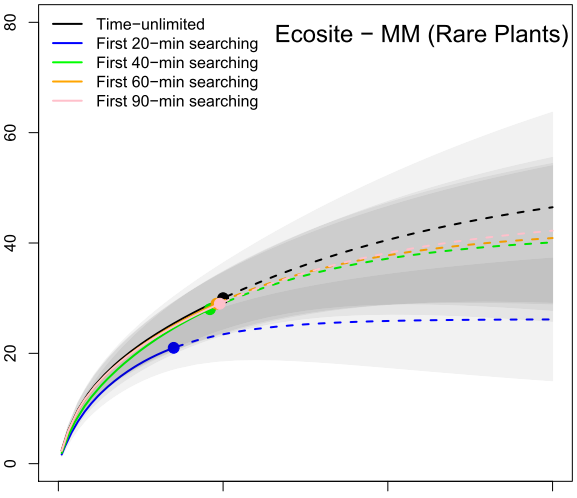

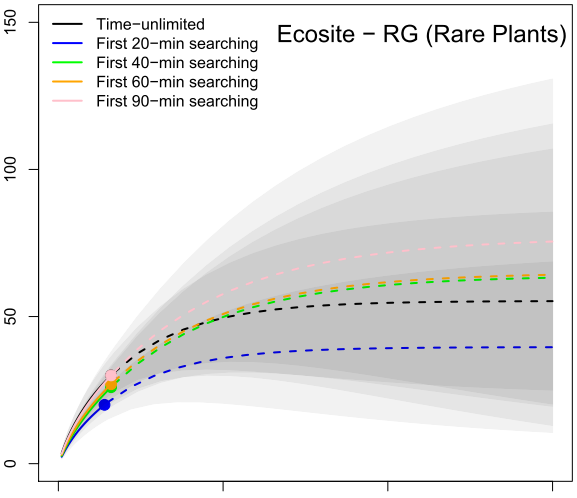

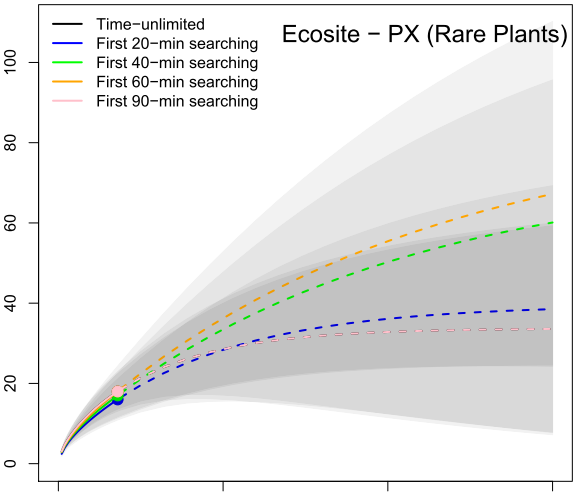


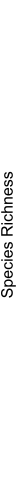

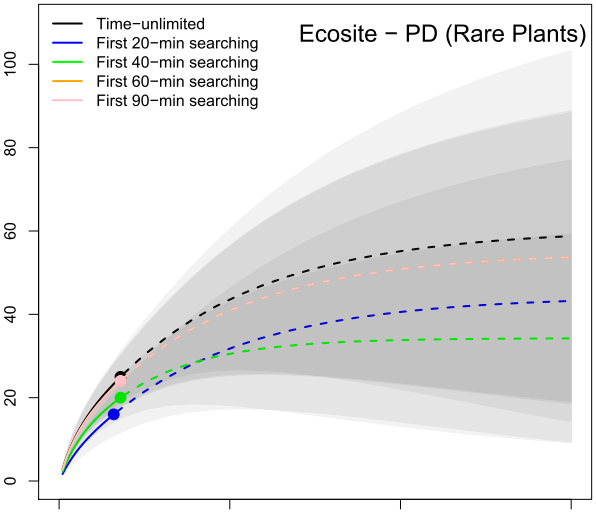

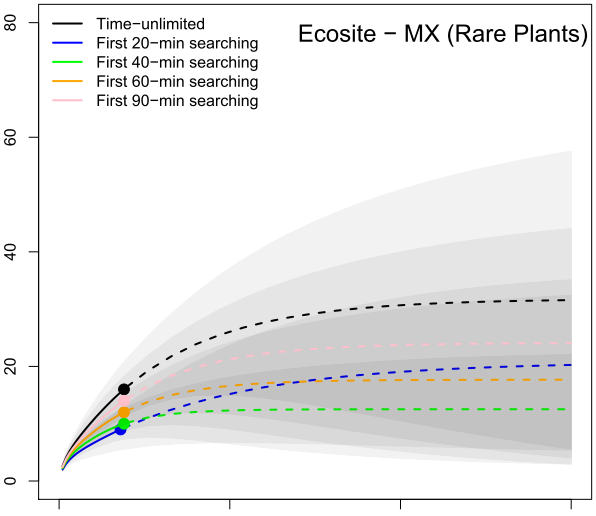

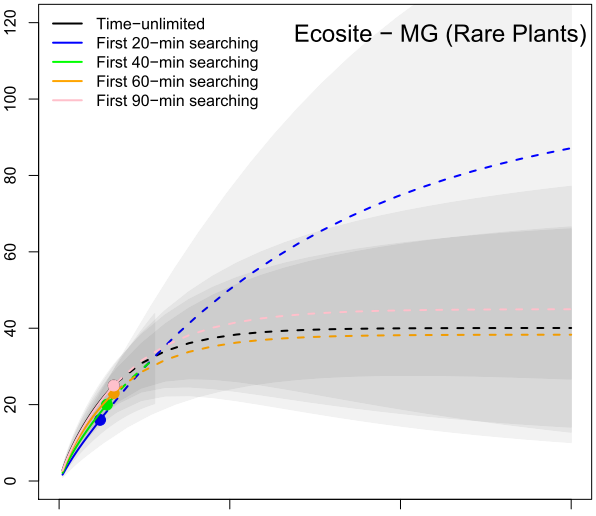


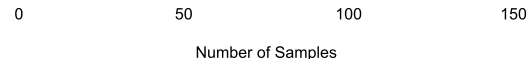

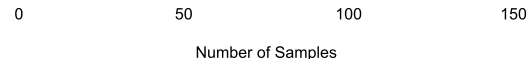

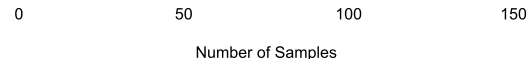

Supplement: Figure S2 — Rarefaction-based species accumulation curves for rare vascular plant species in each of the 9 ecosites sampled. (DOCX) [file pone.0095334.s002.docx]
