# Supplementary material for: Sampling Plant Diversity and Rarity at Landscape Scales: Importance of Sampling Time in Species Detectability
Source: PLoS One. 2014 Apr 16;9(4):e95334. doi: 10.1371/journal.pone.0095334 (PMC3989307; doi:10.1371/journal.pone.0095334)
Supplement: Table S1 — Vascular plant species list in 356 EMCLA plots. NatureServe subnational conservation status: S1: Critically Imperiled; S2: Imperiled; S3: Vulnerable; S4: Apparently Secure; S5: Secure; SNR: Not ranked; SNA: Not applicable; SU: currently unranked. (DOCX) [file pone.0095334.s003.docx]

## Table S1. Vascular plant species list in 356 EMCLA plots. NatureServe subnational conservation status: S1: Critically Imperiled; S2: Imperiled; S3: Vulnerable; S4: Apparently Secure; S5: Secure; SNR: Not ranked; SNA: Not applicable; SU: currently unranked.

| **Scientific name** | **Family** | **NatureServe subnational conservation status** | **Overall prevalence** |
| --- | --- | --- | --- |
| *Lechea intermedia var. depauperata* | Cistaceae | S1 | 1 |
| *Carex adusta* | Cyperaceae | S1 | 1 |
| *Carex hystericina* | Cyperaceae | S1 | 1 |
| *Utricularia cornuta* | Lentibulariaceae | S1 | 1 |
| *Epilobium leptocarpum* | Onagraceae | S1 | 1 |
| *Malaxis paludosa* | Orchidaceae | S1 | 1 |
| *Spiranthes lacera* | Orchidaceae | S1 | 1 |
| *Epilobium glaberrimum* | Onagraceae | S1 | 4 |
| *Hypericum majus* | Clusiaceae | S2 | 1 |
| *Carex umbellata* | Cyperaceae | S2 | 1 |
| *Botrychium simplex* | Ophioglossaceae | S2 | 1 |
| *Lactuca biennis* | Asteraceae | S2 | 2 |
| *Juncus stygius* | Juncaceae | S2 | 2 |
| *Juncus brevicaudatus* | Juncaceae | S2 | 3 |
| *Diphasiastrum sitchense* | Lycopodiaceae | S2 | 5 |
| *Impatiens noli-tangere* | Balsaminaceae | S3 | 1 |
| *Ceratophyllum demersum* | Ceratophyllaceae | S3 | 1 |
| *Carex backii* | Cyperaceae | S3 | 1 |
| *Carex capitata* | Cyperaceae | S3 | 1 |
| *Carex loliacea* | Cyperaceae | S3 | 1 |
| *Eriophorum scheuchzeri* | Cyperaceae | S3 | 1 |
| *Vaccinium uliginosum* | Ericaceae | S3 | 1 |
| *Lycopus asper* | Lamiaceae | S3 | 1 |
| *Goodyera oblongifolia* | Orchidaceae | S3 | 1 |
| *Elymus glaucus* | Poaceae | S3 | 1 |
| *Elymus piperi* | Poaceae | S3 | 1 |
| *Phragmites australis* | Poaceae | S3 | 1 |
| *Sphenopholis intermedia* | Poaceae | S3 | 1 |
| *Viola selkirkii* | Violaceae | S3 | 1 |
| *Carex livida* | Cyperaceae | S3 | 2 |
| *Carex pseudocyperus* | Cyperaceae | S3 | 2 |
| *Carex retrorsa* | Cyperaceae | S3 | 2 |
| *Juncus filiformis* | Juncaceae | S3 | 2 |
| *Botrychium multifidum* | Ophioglossaceae | S3 | 2 |
| *Calamagrostis purpurascens* | Poaceae | S3 | 2 |
| *Polygala senega* | Polygalaceae | S3 | 2 |
| *Salix boothii* | Salicaceae | S3 | 2 |
| *Sparganium minimum* | Sparganiaceae | S3 | 2 |
| *Senecio fremontii* | Asteraceae | S3 | 3 |
| *Eleocharis quinqueflora* | Cyperaceae | S3 | 3 |
| *Drosera linearis* | Droseraceae | S3 | 3 |
| *Dryopteris assimilis* | Dryopteridaceae | S3 | 3 |
| *Lathyrus venosus* | Fabaceae | S3 | 3 |
| *Corallorhiza striata* | Orchidaceae | S3 | 3 |
| *Platanthera dilatata* | Orchidaceae | S3 | 3 |
| *Petasites frigidus var. frigidus* | Asteraceae | S3 | 4 |
| *Cardamine pratensis* | Brassicaceae | S3 | 4 |
| *Hudsonia tomentosa* | Cistaceae | S3 | 4 |
| *Lycopus uniflorus* | Lamiaceae | S3 | 4 |
| *Glyceria pulchella* | Poaceae | S3 | 5 |
| *Carex richardsonii* | Cyperaceae | S3 | 6 |
| *Carex rostrata* | Cyperaceae | S3 | 7 |
| *Drosera anglica* | Droseraceae | S3 | 7 |
| *Gaultheria hispidula* | Ericaceae | S3 | 7 |
| *Monotropa uniflora* | Monotropaceae | S3 | 7 |
| *Corallorhiza maculata* | Orchidaceae | S3 | 7 |
| *Cicuta virosa* | Apiaceae | S3 | 8 |
| *Eriophorum brachyantherum* | Cyperaceae | S3 | 8 |
| *Scirpus cyperinus* | Cyperaceae | S3 | 8 |
| *Juncus vaseyi* | Juncaceae | S3 | 8 |
| *Malaxis monophylla* | Orchidaceae | S3 | 8 |
| *Pyrola elliptica* | Pyrolaceae | S3 | 8 |
| *Sarracenia purpurea* | Sarraceniaceae | S3 | 8 |
| *Pedicularis parviflora* | Scrophulariaceae | S3 | 10 |
| *Carex tenera* | Cyperaceae | S3 | 11 |
| *Carex deflexa* | Cyperaceae | S3 | 13 |
| *Eriophorum chamissonis* | Cyperaceae | S3 | 14 |
| *Carex parryana var. parryana* | Cyperaceae | S3 | 16 |
| *Carex tonsa* | Cyperaceae | S3 | 16 |
| *Scheuchzeria palustris* | Scheuchzeriaceae | S3 | 16 |
| *Carex pauciflora* | Cyperaceae | S3 | 19 |
| *Eriophorum gracile* | Cyperaceae | S3 | 19 |
| *Coptis trifolia* | Ranunculaceae | S3 | 19 |
| *Salix athabascensis* | Salicaceae | S3 | 19 |
| *Carex prairea* | Cyperaceae | S3 | 20 |
| *Carex interior* | Cyperaceae | S3 | 21 |
| *Epilobium leptophyllum* | Onagraceae | S3 | 26 |
| *Lysimachia thyrsiflora* | Primulaceae | S3 | 28 |
| *Carex trisperma* | Cyperaceae | S3 | 29 |
| *Lycopodium clavatum* | Lycopodiaceae | S3 | 29 |
| *Platanthera orbiculata* | Orchidaceae | S3 | 43 |
| *Galium labradoricum* | Rubiaceae | S3 | 43 |
| *Rhamnus alnifolia* | Rhamnaceae | S3 | 46 |
| *Kalmia polifolia* | Ericaceae | S3 | 48 |
| *Epilobium palustre* | Onagraceae | S3 | 55 |
| *Potentilla tridentata* | Rosaceae | S3 | 61 |
| *Melampyrum lineare* | Scrophulariaceae | S3 | 66 |
| *Lonicera caerulea* | Caprifoliaceae | S3 | 111 |
| *Carex oligosperma* | Cyperaceae | S3? | 7 |
| *Chrysosplenium iowense* | Saxifragaceae | S3? | 13 |
| *Carex viridula* | Cyperaceae | S3S4 | 1 |
| *Anemone riparia* | Ranunculaceae | S3S4 | 1 |
| *Carex houghtoniana* | Cyperaceae | S3S4 | 2 |
| *Potamogeton alpinus* | Potamogetonaceae | S3S4 | 3 |
| *Chrysosplenium tetrandrum* | Saxifragaceae | S3S4 | 3 |
| *Myrica gale* | Myricaceae | S3S4 | 9 |
| *Carex tenuiflora* | Cyperaceae | S3S4 | 47 |
| *Symphyotrichum ericoides* | Asteraceae | S4 | 1 |
| *Cerastium nutans* | Caryophyllaceae | S4 | 1 |
| *Carex microptera* | Cyperaceae | S4 | 1 |
| *Carex rossii* | Cyperaceae | S4 | 1 |
| *Scirpus cespitosus* | Cyperaceae | S4 | 1 |
| *Scirpus hudsonianus* | Cyperaceae | S4 | 1 |
| *Spirodela polyrrhiza* | Lemnaceae | S4 | 1 |
| *Nuphar lutea* | Nymphaeaceae | S4 | 1 |
| *Cypripedium passerinum* | Orchidaceae | S4 | 1 |
| *Agropyron smithii* | Poaceae | S4 | 1 |
| *Glyceria borealis* | Poaceae | S4 | 1 |
| *Muhlenbergia cuspidata* | Poaceae | S4 | 1 |
| *Polygonum coccineum* | Polygonaceae | S4 | 1 |
| *Glaux maritima* | Primulaceae | S4 | 1 |
| *Agrimonia striata* | Rosaceae | S4 | 1 |
| *Ribes americanum* | Grossulariaceae | S4 | 2 |
| *Festuca rubra* | Poaceae | S4 | 2 |
| *Muhlenbergia glomerata* | Poaceae | S4 | 2 |
| *Lysimachia ciliata* | Primulaceae | S4 | 2 |
| *Arceuthobium americanum* | Viscaceae | S4 | 2 |
| *Erigeron elatus* | Asteraceae | S4 | 3 |
| *Solidago nemoralis* | Asteraceae | S4 | 3 |
| *Schoenoplectus acutus* | Cyperaceae | S4 | 3 |
| *Phacelia franklinii* | Hydrophyllaceae | S4 | 3 |
| *Agastache foeniculum* | Lamiaceae | S4 | 3 |
| *Listera borealis* | Orchidaceae | S4 | 3 |
| *Elymus canadensis* | Poaceae | S4 | 3 |
| *Scolochloa festucacea* | Poaceae | S4 | 3 |
| *Potamogeton gramineus* | Potamogetonaceae | S4 | 3 |
| *Arabis lyrata* | Brassicaceae | S4 | 4 |
| *Polemonium acutiflorum* | Polemoniaceae | S4 | 4 |
| *Salix glauca* | Salicaceae | S4 | 4 |
| *Ribes hirtellum* | Grossulariaceae | S4 | 5 |
| *Sanicula marilandica* | Apiaceae | S4 | 6 |
| *Calla palustris* | Araceae | S4 | 6 |
| *Symphyotrichum puniceum var. puniceum* | Asteraceae | S4 | 6 |
| *Carex peckii* | Cyperaceae | S4 | 6 |
| *Pyrola minor* | Pyrolaceae | S4 | 6 |
| *Carex aenea* | Cyperaceae | S4 | 7 |
| *Eriophorum viridi-carinatum* | Cyperaceae | S4 | 7 |
| *Sorbus scopulina* | Rosaceae | S4 | 7 |
| *Rhinanthus minor* | Scrophulariaceae | S4 | 7 |
| *Carex bebbii* | Cyperaceae | S4 | 8 |
| *Impatiens capensis* | Balsaminaceae | S4 | 9 |
| *Betula occidentalis* | Betulaceae | S4 | 9 |
| *Viola palustris* | Violaceae | S4 | 11 |
| *Halenia deflexa* | Gentianaceae | S4 | 12 |
| *Utricularia minor* | Lentibulariaceae | S4 | 13 |
| *Salix arbusculoides* | Salicaceae | S4 | 14 |
| *Utricularia intermedia* | Lentibulariaceae | S4 | 16 |
| *Carex deweyana* | Cyperaceae | S4 | 18 |
| *Circaea alpina* | Onagraceae | S4 | 18 |
| *Cicuta bulbifera* | Apiaceae | S4 | 20 |
| *Carex lasiocarpa* | Cyperaceae | S4 | 22 |
| *Carex sartwellii* | Cyperaceae | S4 | 22 |
| *Dryopteris carthusiana* | Dryopteridaceae | S4 | 23 |
| *Listera cordata* | Orchidaceae | S4 | 23 |
| *Eriophorum angustifolium* | Cyperaceae | S4 | 24 |
| *Viola nephrophylla* | Violaceae | S4 | 24 |
| *Glyceria striata* | Poaceae | S4 | 26 |
| *Fragaria vesca* | Rosaceae | S4 | 27 |
| *Salix candida* | Salicaceae | S4 | 27 |
| *Salix pseudomonticola* | Salicaceae | S4 | 27 |
| *Salix scouleriana* | Salicaceae | S4 | 29 |
| *Cinna latifolia* | Poaceae | S4 | 30 |
| *Betula papyrifera* | Betulaceae | S4 | 31 |
| *Salix serissima* | Salicaceae | S4 | 36 |
| *Symphyotrichum puniceum* | Asteraceae | S4 | 39 |
| *Calamagrostis stricta* | Poaceae | S4 | 39 |
| *Ranunculus lapponicus* | Ranunculaceae | S4 | 39 |
| *Carex limosa* | Cyperaceae | S4 | 44 |
| *Lycopodium obscurum* | Lycopodiaceae | S4 | 48 |
| *Carex brunnescens* | Cyperaceae | S4 | 57 |
| *Salix pyrifolia* | Salicaceae | S4 | 66 |
| *Oryzopsis asperifolia* | Poaceae | S4 | 68 |
| *Salix pedicellaris* | Salicaceae | S4 | 73 |
| *Chamaedaphne calyculata* | Ericaceae | S4 | 74 |
| *Carex paupercula* | Cyperaceae | S4 | 77 |
| *Oryzopsis pungens* | Poaceae | S4 | 81 |
| *Trientalis borealis* | Primulaceae | S4 | 179 |
| *Danthonia intermedia* | Poaceae | S4S5 | 3 |
| *Botrychium virginianum* | Ophioglossaceae | S4S5 | 10 |
| *Elymus trachycaulus ssp. subsecundus* | Poaceae | S4S5 | 10 |
| *Alnus viridis ssp. crispa* | Betulaceae | S4S5 | 21 |
| *Zizia aptera* | Apiaceae | S5 | 1 |
| *Antennaria parvifolia* | Asteraceae | S5 | 1 |
| *Antennaria rosea* | Asteraceae | S5 | 1 |
| *Cirsium drummondii* | Asteraceae | S5 | 1 |
| *Erigeron lonchophyllus* | Asteraceae | S5 | 1 |
| *Euthamia graminifolia* | Asteraceae | S5 | 1 |
| *Solidago gigantea* | Asteraceae | S5 | 1 |
| *Arabis divaricarpa* | Brassicaceae | S5 | 1 |
| *Lepidium densiflorum* | Brassicaceae | S5 | 1 |
| *Callitriche verna* | Callitrichaceae | S5 | 1 |
| *Cerastium arvense* | Caryophyllaceae | S5 | 1 |
| *Stellaria crassifolia* | Caryophyllaceae | S5 | 1 |
| *Carex canescens ssp. canescens* | Cyperaceae | S5 | 1 |
| *Carex praegracilis* | Cyperaceae | S5 | 1 |
| *Eleocharis acicularis* | Cyperaceae | S5 | 1 |
| *Kalmia microphylla* | Ericaceae | S5 | 1 |
| *Oxytropis deflexa* | Fabaceae | S5 | 1 |
| *Oxytropis splendens* | Fabaceae | S5 | 1 |
| *Juncus alpinoarticulatus* | Juncaceae | S5 | 1 |
| *Juncus bufonius* | Juncaceae | S5 | 1 |
| *Juncus nodosus* | Juncaceae | S5 | 1 |
| *Beckmannia syzigachne* | Poaceae | S5 | 1 |
| *Rumex triangulivalvis* | Polygonaceae | S5 | 1 |
| *Potamogeton richardsonii* | Potamogetonaceae | S5 | 1 |
| *Anemone parviflora* | Ranunculaceae | S5 | 1 |
| *Caltha natans* | Ranunculaceae | S5 | 1 |
| *Ranunculus cymbalaria* | Ranunculaceae | S5 | 1 |
| *Potentilla anserina* | Rosaceae | S5 | 1 |
| *Potentilla fruticosa* | Rosaceae | S5 | 1 |
| *Potentilla gracilis* | Rosaceae | S5 | 1 |
| *Spiraea betulifolia* | Rosaceae | S5 | 1 |
| *Salix lucida* | Salicaceae | S5 | 1 |
| *Arnica chamissonis* | Asteraceae | S5 | 2 |
| *Senecio congestus* | Asteraceae | S5 | 2 |
| *Solidago simplex ssp. simplex* | Asteraceae | S5 | 2 |
| *Symphyotrichum falcatum* | Asteraceae | S5 | 2 |
| *Rorippa palustris* | Brassicaceae | S5 | 2 |
| *Carex crawfordii* | Cyperaceae | S5 | 2 |
| *Carex pellita* | Cyperaceae | S5 | 2 |
| *Corydalis aurea* | Fumariaceae | S5 | 2 |
| *Allium cernuum* | Liliaceae | S5 | 2 |
| *Streptopus amplexifolius* | Liliaceae | S5 | 2 |
| *Bromus inermis* | Poaceae | S5 | 2 |
| *Potamogeton pusillus* | Potamogetonaceae | S5 | 2 |
| *Primula incana* | Primulaceae | S5 | 2 |
| *Anemone cylindrica* | Ranunculaceae | S5 | 2 |
| *Ranunculus macounii* | Ranunculaceae | S5 | 2 |
| *Heuchera richardsonii* | Saxifragaceae | S5 | 2 |
| *Heracleum lanatum* | Apiaceae | S5 | 3 |
| *Canadanthus modestus* | Asteraceae | S5 | 3 |
| *Erigeron glabellus* | Asteraceae | S5 | 3 |
| *Erysimum cheiranthoides* | Brassicaceae | S5 | 3 |
| *Carex praticola* | Cyperaceae | S5 | 3 |
| *Schoenoplectus tabernaemontani* | Cyperaceae | S5 | 3 |
| *Scirpus microcarpus* | Cyperaceae | S5 | 3 |
| *Myriophyllum exalbescens* | Haloragaceae | S5 | 3 |
| *Juncus tenuis* | Juncaceae | S5 | 3 |
| *Calypso bulbosa* | Orchidaceae | S5 | 3 |
| *Coeloglossum viride* | Orchidaceae | S5 | 3 |
| *Phalaris arundinacea* | Poaceae | S5 | 3 |
| *Ranunculus abortivus* | Ranunculaceae | S5 | 3 |
| *Ranunculus aquatilis* | Ranunculaceae | S5 | 3 |
| *Veronica americana* | Scrophulariaceae | S5 | 3 |
| *Erigeron philadelphicus* | Asteraceae | S5 | 4 |
| *Senecio eremophilus* | Asteraceae | S5 | 4 |
| *Triglochin palustris* | Juncaginaceae | S5 | 4 |
| *Alopecurus aequalis* | Poaceae | S5 | 4 |
| *Hordeum jubatum* | Poaceae | S5 | 4 |
| *Sparganium angustifolium* | Sparganiaceae | S5 | 4 |
| *Bidens cernua* | Asteraceae | S5 | 5 |
| *Symphoricarpos occidentalis* | Caprifoliaceae | S5 | 5 |
| *Equisetum variegatum* | Equisetaceae | S5 | 5 |
| *Sisyrinchium montanum* | Iridaceae | S5 | 5 |
| *Tofieldia glutinosa* | Liliaceae | S5 | 5 |
| *Glyceria grandis* | Poaceae | S5 | 5 |
| *Koeleria macrantha* | Poaceae | S5 | 5 |
| *Aquilegia brevistyla* | Ranunculaceae | S5 | 5 |
| *Cicuta maculata* | Apiaceae | S5 | 6 |
| *Erigeron acris* | Asteraceae | S5 | 6 |
| *Symphyotrichum lanceolatum var. hesperium* | Asteraceae | S5 | 6 |
| *Stellaria calycantha* | Caryophyllaceae | S5 | 6 |
| *Equisetum palustre* | Equisetaceae | S5 | 6 |
| *Hedysarum alpinum* | Fabaceae | S5 | 6 |
| *Anemone canadensis* | Ranunculaceae | S5 | 6 |
| *Delphinium glaucum* | Ranunculaceae | S5 | 6 |
| *Sium suave* | Apiaceae | S5 | 7 |
| *Achillea sibirica* | Asteraceae | S5 | 7 |
| *Erigeron canadensis* | Asteraceae | S5 | 7 |
| *Cardamine pensylvanica* | Brassicaceae | S5 | 7 |
| *Empetrum nigrum* | Empetraceae | S5 | 7 |
| *Ranunculus sceleratus* | Ranunculaceae | S5 | 7 |
| *Arctostaphylos rubra* | Ericaceae | S5 | 8 |
| *Corydalis sempervirens* | Fumariaceae | S5 | 8 |
| *Dracocephalum parviflorum* | Lamiaceae | S5 | 8 |
| *Petasites frigidus var. x vitifolius* | Asteraceae | S5 | 9 |
| *Stachys palustris* | Lamiaceae | S5 | 9 |
| *Elymus trachycaulus ssp. trachycaulus* | Poaceae | S5 | 9 |
| *Osmorhiza depauperata* | Apiaceae | S5 | 10 |
| *Carex concinna* | Cyperaceae | S5 | 10 |
| *Eleocharis palustris* | Cyperaceae | S5 | 10 |
| *Hippuris vulgaris* | Hippuridaceae | S5 | 10 |
| *Geum rivale* | Rosaceae | S5 | 10 |
| *Antennaria neglecta* | Asteraceae | S5 | 11 |
| *Stellaria longipes* | Caryophyllaceae | S5 | 11 |
| *Carex norvegica* | Cyperaceae | S5 | 11 |
| *Gymnocarpium dryopteris* | Dryopteridaceae | S5 | 11 |
| *Utricularia vulgaris* | Lentibulariaceae | S5 | 11 |
| *Artemisia campestris* | Asteraceae | S5 | 12 |
| *Adoxa moschatellina* | Adoxaceae | S5 | 13 |
| *Carex capillaris* | Cyperaceae | S5 | 13 |
| *Luzula parviflora* | Juncaceae | S5 | 13 |
| *Smilacina stellata* | Liliaceae | S5 | 13 |
| *Symphyotrichum boreale* | Asteraceae | S5 | 14 |
| *Lemna minor* | Lemnaceae | S5 | 14 |
| *Anemone patens* | Ranunculaceae | S5 | 14 |
| *Solidago multiradiata* | Asteraceae | S5 | 15 |
| *Alnus incana* | Betulaceae | S5 | 15 |
| *Betula glandulosa* | Betulaceae | S5 | 15 |
| *Carex aurea* | Cyperaceae | S5 | 15 |
| *Astragalus americanus* | Fabaceae | S5 | 15 |
| *Festuca saximontana* | Poaceae | S5 | 15 |
| *Solidago missouriensis* | Asteraceae | S5 | 16 |
| *Deschampsia cespitosa* | Poaceae | S5 | 16 |
| *Carex atherodes* | Cyperaceae | S5 | 18 |
| *Gentianella amarella* | Gentianaceae | S5 | 18 |
| *Mentha arvensis* | Lamiaceae | S5 | 18 |
| *Disporum trachycarpum* | Liliaceae | S5 | 19 |
| *Anemone multifida* | Ranunculaceae | S5 | 19 |
| *Geum macrophyllum* | Rosaceae | S5 | 19 |
| *Apocynum androsaemifolium* | Apocynaceae | S5 | 20 |
| *Corylus cornuta* | Betulaceae | S5 | 20 |
| *Platanthera obtusata* | Orchidaceae | S5 | 20 |
| *Solidago simplex* | Asteraceae | S5 | 21 |
| *Amerorchis rotundifolia* | Orchidaceae | S5 | 21 |
| *Eurybia conspicua* | Asteraceae | S5 | 22 |
| *Polygonum amphibium* | Polygonaceae | S5 | 22 |
| *Petasites frigidus* | Asteraceae | S5 | 23 |
| *Salix petiolaris* | Salicaceae | S5 | 23 |
| *Typha latifolia* | Typhaceae | S5 | 23 |
| *Moehringia lateriflora* | Caryophyllaceae | S5 | 24 |
| *Juncus balticus* | Juncaceae | S5 | 24 |
| *Ranunculus gmelinii* | Ranunculaceae | S5 | 24 |
| *Thalictrum venulosum* | Ranunculaceae | S5 | 24 |
| *Geum aleppicum* | Rosaceae | S5 | 25 |
| *Prunus virginiana* | Rosaceae | S5 | 25 |
| *Senecio pauperculus* | Asteraceae | S5 | 26 |
| *Cornus stolonifera* | Cornaceae | S5 | 28 |
| *Geranium bicknellii* | Geraniaceae | S5 | 28 |
| *Salix discolor* | Salicaceae | S5 | 28 |
| *Calamagrostis inexpansa* | Poaceae | S5 | 29 |
| *Viola canadensis* | Violaceae | S5 | 29 |
| *Moneses uniflora* | Pyrolaceae | S5 | 30 |
| *Lilium philadelphicum* | Liliaceae | S5 | 31 |
| *Elymus trachycaulus* | Poaceae | S5 | 32 |
| *Menyanthes trifoliata* | Menyanthaceae | S5 | 34 |
| *Pedicularis labradorica* | Scrophulariaceae | S5 | 34 |
| *Rosa woodsii* | Rosaceae | S5 | 35 |
| *Urtica dioica* | Urticaceae | S5 | 35 |
| *Poa palustris* | Poaceae | S5 | 36 |
| *Potentilla norvegica* | Rosaceae | S5 | 36 |
| *Symphyotrichum laeve* | Asteraceae | S5 | 37 |
| *Triglochin maritima* | Juncaginaceae | S5 | 37 |
| *Carex chordorrhiza* | Cyperaceae | S5 | 38 |
| *Corallorhiza trifida* | Orchidaceae | S5 | 38 |
| *Comandra umbellata* | Santalaceae | S5 | 38 |
| *Scutellaria galericulata* | Lamiaceae | S5 | 40 |
| *Carex leptalea* | Cyperaceae | S5 | 41 |
| *Prunus pensylvanica* | Rosaceae | S5 | 42 |
| *Alnus incana ssp. tenuifolia* | Betulaceae | S5 | 43 |
| *Vaccinium caespitosum* | Ericaceae | S5 | 44 |
| *Epilobium ciliatum* | Onagraceae | S5 | 45 |
| *Abies balsamea* | Pinaceae | S5 | 45 |
| *Viola adunca* | Violaceae | S5 | 45 |
| *Bromus ciliatus* | Poaceae | S5 | 46 |
| *Galium trifidum* | Rubiaceae | S5 | 48 |
| *Equisetum pratense* | Equisetaceae | S5 | 49 |
| *Parnassia palustris* | Saxifragaceae | S5 | 49 |
| *Goodyera repens* | Orchidaceae | S5 | 50 |
| *Spiranthes romanzoffiana* | Orchidaceae | S5 | 50 |
| *Carex diandra* | Cyperaceae | S5 | 51 |
| *Ribes glandulosum* | Grossulariaceae | S5 | 53 |
| *Poa pratensis* | Poaceae | S5 | 54 |
| *Ribes lacustre* | Grossulariaceae | S5 | 55 |
| *Salix maccalliana* | Salicaceae | S5 | 56 |
| *Rumex occidentalis* | Polygonaceae | S5 | 58 |
| *Schizachne purpurascens* | Poaceae | S5 | 60 |
| *Pyrola chlorantha* | Pyrolaceae | S5 | 60 |
| *Petasites frigidus var. sagittatus* | Asteraceae | S5 | 61 |
| *Carex canescens* | Cyperaceae | S5 | 62 |
| *Carex vaginata* | Cyperaceae | S5 | 63 |
| *Eriophorum vaginatum* | Cyperaceae | S5 | 63 |
| *Symphoricarpos albus* | Caprifoliaceae | S5 | 64 |
| *Carex utriculata* | Cyperaceae | S5 | 64 |
| *Equisetum fluviatile* | Equisetaceae | S5 | 65 |
| *Andromeda polifolia* | Ericaceae | S5 | 68 |
| *Platanthera hyperborea* | Orchidaceae | S5 | 68 |
| *Oxycoccus microcarpus* | Ericaceae | S5 | 69 |
| *Diphasiastrum complanatum* | Lycopodiaceae | S5 | 69 |
| *Lonicera involucrata* | Caprifoliaceae | S5 | 73 |
| *Ribes hudsonianum* | Grossulariaceae | S5 | 74 |
| *Actaea rubra* | Ranunculaceae | S5 | 74 |
| *Galium triflorum* | Rubiaceae | S5 | 74 |
| *Drosera rotundifolia* | Droseraceae | S5 | 76 |
| *Carex gynocrates* | Cyperaceae | S5 | 79 |
| *Caltha palustris* | Ranunculaceae | S5 | 79 |
| *Alnus viridis* | Betulaceae | S5 | 80 |
| *Lycopodium annotinum* | Lycopodiaceae | S5 | 80 |
| *Agrostis scabra* | Poaceae | S5 | 80 |
| *Carex siccata* | Cyperaceae | S5 | 83 |
| *Shepherdia canadensis* | Elaeagnaceae | S5 | 85 |
| *Salix myrtillifolia* | Salicaceae | S5 | 85 |
| *Equisetum scirpoides* | Equisetaceae | S5 | 87 |
| *Hieracium umbellatum* | Asteraceae | S5 | 90 |
| *Campanula rotundifolia* | Campanulaceae | S5 | 92 |
| *Potentilla palustris* | Rosaceae | S5 | 93 |
| *Vicia americana* | Fabaceae | S5 | 95 |
| *Stellaria longifolia* | Caryophyllaceae | S5 | 97 |
| *Ribes oxyacanthoides* | Grossulariaceae | S5 | 97 |
| *Rubus arcticus* | Rosaceae | S5 | 98 |
| *Lonicera dioica* | Caprifoliaceae | S5 | 100 |
| *Mertensia paniculata* | Boraginaceae | S5 | 102 |
| *Rubus chamaemorus* | Rosaceae | S5 | 106 |
| *Lathyrus ochroleucus* | Fabaceae | S5 | 108 |
| *Amelanchier alnifolia* | Rosaceae | S5 | 110 |
| *Ribes triste* | Grossulariaceae | S5 | 114 |
| *Geocaulon lividum* | Santalaceae | S5 | 115 |
| *Arctostaphylos uva-ursi* | Ericaceae | S5 | 116 |
| *Viola renifolia* | Violaceae | S5 | 118 |
| *Aralia nudicaulis* | Araliaceae | S5 | 119 |
| *Salix planifolia* | Salicaceae | S5 | 119 |
| *Populus balsamifera* | Salicaceae | S5 | 121 |
| *Pinus banksiana* | Pinaceae | S5 | 128 |
| *Galium boreale* | Rubiaceae | S5 | 130 |
| *Leymus innovatus* | Poaceae | S5 | 131 |
| *Viburnum edule* | Caprifoliaceae | S5 | 132 |
| *Symphyotrichum ciliolatum* | Asteraceae | S5 | 138 |
| *Carex disperma* | Cyperaceae | S5 | 139 |
| *Rubus idaeus* | Rosaceae | S5 | 140 |
| *Rubus pubescens* | Rosaceae | S5 | 141 |
| *Betula pumila* | Betulaceae | S5 | 146 |
| *Achillea millefolium* | Asteraceae | S5 | 150 |
| *Larix laricina* | Pinaceae | S5 | 150 |
| *Fragaria virginiana* | Rosaceae | S5 | 156 |
| *Mitella nuda* | Saxifragaceae | S5 | 156 |
| *Pyrola asarifolia* | Pyrolaceae | S5 | 157 |
| *Petasites frigidus var. palmatus* | Asteraceae | S5 | 163 |
| *Carex aquatilis* | Cyperaceae | S5 | 163 |
| *Equisetum arvense* | Equisetaceae | S5 | 163 |
| *Picea glauca* | Pinaceae | S5 | 167 |
| *Smilacina trifolia* | Liliaceae | S5 | 169 |
| *Equisetum sylvaticum* | Equisetaceae | S5 | 172 |
| *Betula neoalaskana* | Betulaceae | S5 | 173 |
| *Maianthemum canadense* | Liliaceae | S5 | 174 |
| *Orthilia secunda* | Pyrolaceae | S5 | 195 |
| *Populus tremuloides* | Salicaceae | S5 | 200 |
| *Salix bebbiana* | Salicaceae | S5 | 208 |
| *Epilobium angustifolium* | Onagraceae | S5 | 213 |
| *Vaccinium myrtilloides* | Ericaceae | S5 | 217 |
| *Linnaea borealis* | Caprifoliaceae | S5 | 223 |
| *Rosa acicularis* | Rosaceae | S5 | 227 |
| *Calamagrostis canadensis* | Poaceae | S5 | 229 |
| *Cornus canadensis* | Cornaceae | S5 | 235 |
| *Picea mariana* | Pinaceae | S5 | 240 |
| *Ledum groenlandicum* | Ericaceae | S5 | 278 |
| *Vaccinium vitis-idaea* | Ericaceae | S5 | 288 |
| *Equisetum hyemale* | Equisetaceae | S5? | 19 |
| *Cirsium vulgare* | Asteraceae | SNA | 1 |
| *Hieracium caespitosum* | Asteraceae | SNA | 1 |
| *Sonchus oleraceus* | Asteraceae | SNA | 1 |
| *Capsella bursa-pastoris* | Brassicaceae | SNA | 1 |
| *Nasturtium officinale* | Brassicaceae | SNA | 1 |
| *Cerastium fontanum ssp. vulgare* | Caryophyllaceae | SNA | 1 |
| *Chenopodium album* | Chenopodiaceae | SNA | 1 |
| *Astragalus cicer* | Fabaceae | SNA | 1 |
| *Melilotus officinalis* | Fabaceae | SNA | 1 |
| *Bromus inermis ssp. inermis* | Poaceae | SNA | 1 |
| *Phalaris canariensis* | Poaceae | SNA | 1 |
| *Hieracium aurantiacum* | Asteraceae | SNA | 2 |
| *Stellaria media* | Caryophyllaceae | SNA | 2 |
| *Medicago lupulina* | Fabaceae | SNA | 2 |
| *Agrostis stolonifera* | Poaceae | SNA | 2 |
| *Elytrigia repens* | Poaceae | SNA | 2 |
| *Phleum pratense* | Poaceae | SNA | 2 |
| *Polygonum persicaria* | Polygonaceae | SNA | 2 |
| *Sonchus uliginosus* | Asteraceae | SNA | 3 |
| *Melilotus alba* | Fabaceae | SNA | 3 |
| *Chamaesaracha grandiflora* | Solanaceae | SNA | 3 |
| *Medicago sativa* | Fabaceae | SNA | 4 |
| *Euphrasia nemorosa* | Scrophulariaceae | SNA | 4 |
| *Plantago major* | Plantaginaceae | SNA | 6 |
| *Trifolium repens* | Fabaceae | SNA | 7 |
| *Galeopsis tetrahit* | Lamiaceae | SNA | 9 |
| *Cirsium arvense* | Asteraceae | SNA | 12 |
| *Trifolium pratense* | Fabaceae | SNA | 12 |
| *Trifolium hybridum* | Fabaceae | SNA | 14 |
| *Sonchus arvensis* | Asteraceae | SNA | 18 |
| *Crepis tectorum* | Asteraceae | SNA | 33 |
| *Taraxacum officinale* | Asteraceae | SNA | 66 |
| *Erigeron acris ssp. politus* | Asteraceae | SNR | 1 |
| *Symphyotrichum laeve var. laeve* | Asteraceae | SNR | 1 |
| *Arabis holboellii var. collinsii* | Brassicaceae | SNR | 1 |
| *Silene latifolia* | Caryophyllaceae | SNR | 1 |
| *Chenopodium leptophyllum* | Chenopodiaceae | SNR | 1 |
| *Solidago simplex var. simplex* | Asteraceae | SNR | 2 |
| *Callitriche palustris* | Callitrichaceae | SNR | 2 |
| *Equisetum hyemale ssp. affine* | Equisetaceae | SNR | 2 |
| *Cypripedium parviflorum* | Orchidaceae | SNR | 2 |
| *Antennaria microphylla* | Asteraceae | SNR | 3 |
| *Dryopteris expansa* | Dryopteridaceae | SNR | 3 |
| *Hierochloe hirta* | Poaceae | SNR | 3 |
| *Salix lucida ssp. lasiandra* | Salicaceae | SNR | 3 |
| *Lycopodium dendroideum* | Lycopodiaceae | SNR | 4 |
| *Cornus sericea ssp. sericea* | Cornaceae | SNR | 18 |
| *Salix myrtillifolia var. cordata* | Salicaceae | SNR | 19 |
| *Solidago altissima* | Asteraceae | SNR | 21 |
| *Chamerion angustifolium ssp. angustifolium* | Onagraceae | SNR | 23 |
| *Vaccinium oxycoccos* | Ericaceae | SNR | 89 |
| *Panicum acuminatum* | Poaceae | SU | 1 |
