# Supplementary material for: Sampling Plant Diversity and Rarity at Landscape Scales: Importance of Sampling Time in Species Detectability
Source: PLoS One. 2014 Apr 16;9(4):e95334. doi: 10.1371/journal.pone.0095334 (PMC3989307; doi:10.1371/journal.pone.0095334)
Supplement: Table S2 — Comparison of observed vascular plant species richness between ABMI and EMCLA plots in each of four 0.25-hectare quadrats at four ABMI one-hectare sites. (DOCX) [file pone.0095334.s004.docx]

## Table S2. Comparison of observed vascular plant species richness between ABMI and EMCLA plots in each of four 0.25-hectare quadrats at four ABMI one-hectare sites.

|  |  | Total plant richness | | Rare plant richness | |
| --- | --- | --- | --- | --- | --- |
| Site | **Quadrats** | **ABMI** | **EMCLA** | **ABMI** | **EMCLA** |
| A | NE | 40 | 71 | 0 | 2 |
|  | NW | 39 | 62 | 0 | 1 |
|  | SE | 41 | 84 | 0 | 4 |
|  | SW | 43 | 73 | 0 | 2 |
|  |  |  |  |  |  |
| B | NE | 39 | 52 | 0 | 3 |
|  | NW | 41 | 52 | 1 | 2 |
|  | SE | 39 | 57 | 0 | 1 |
|  | SW | 39 | 54 | 1 | 2 |
|  |  |  |  |  |  |
| C | NE | 34 | 44 | 0 | 2 |
|  | NW | 34 | 50 | 0 | 1 |
|  | SE | 35 | 51 | 0 | 0 |
|  | SW | 38 | 52 | 0 | 1 |
|  |  |  |  |  |  |
| D | NE | 39 | 73 | 0 | 2 |
|  | NW | 36 | 43 | 0 | 2 |
|  | SE | 38 | 73 | 0 | 2 |
|  | SW | 35 | 58 | 0 | 0 |
